# In silico drug discovery and molecular dynamics simulation for targeting neonatal pneumonia and bronchopulmonary dysplasia

**DOI:** 10.3389/fchem.2026.1859262

**Published:** 2026-06-30

**Authors:** Xiaoqin Chen, Qian Liu, Bing Zhang

**Affiliations:** Xinxiang Central Hospital (Henan Medical University Fourth Clinical College), Neonatal Intensive Care Unit, Xinxiang, Henan, China

**Keywords:** bronchopulmonary dysplasia, *in silico* drug discovery, molecular dynamics simulation, neonatal pneumonia, NLRP3 inflammasome, traditional Chinese medicine

## Abstract

**Background:**

Neonatal pneumonia and bronchopulmonary dysplasia (BPD) are major causes of morbidity and mortality in preterm infants, driven by excessive inflammation involving the NOD-like receptor family pyrin domain-containing 3 (NLRP3) inflammasome. This study employed *in silico* drug discovery, including virtual screening, molecular docking, ADMET profiling, molecular dynamics (MD) simulations, and MM/PBSA calculations, followed by preliminary *in vitro* validation to identify novel NLRP3 inhibitors from Traditional Chinese Medicine (TCM) compounds for these conditions.

**Methods:**

The NLRP3 NACHT domain (PDB ID: 7ALV) served as the target. A library of FDA-approved drugs and TCM-derived compounds underwent molecular docking with AutoDock Vina. Top hits were evaluated for ADMET properties using SwissADME, pkCSM, and admetSAR. Selected complexes (Hinokiflavone, Theaflavin, Sciadopitysin, Liquiritin apioside, Tigogenin) were subjected to 200 ns all-atom MD simulations in GROMACS and MM/PBSA binding free energy analysis. *In vitro* cytoprotective effects were assessed via MTT assay in LPS-stimulated BEAS-2B and MLE-12 lung epithelial cells, with glyburide as positive control.

**Results:**

Hinokiflavone and Theaflavin exhibited the strongest docking scores (−10.8 and −10.4 kcal/mol), superior MD stability (lowest RMSD: 0.21 ± 0.02 nm and 0.23 ± 0.03 nm; high hydrogen bond occupancy: 78% and 82%), and most favorable MM/PBSA binding energies (−55.8 and −55.2 kcal/mol), driven by van der Waals and electrostatic interactions. They showed acceptable ADMET profiles with high intestinal absorption and low BBB penetration. *In vitro*, Hinokiflavone restored cell viability to 89.6% ± 3.2% at 50 µM (comparable to glyburide at 91.2% ± 2.8%), while Theaflavin reached 82.4% ± 3.9%, demonstrating dose-dependent protection against LPS-induced cytotoxicity.

**Conclusion:**

Hinokiflavone and Theaflavin emerge as promising NLRP3 inhibitors with stable binding to the NACHT domain and cytoprotective effects in lung epithelial cells. These TCM-derived compounds warrant further preclinical investigation as potential targeted therapies to mitigate inflammation in neonatal pneumonia and BPD.

## 1 Introduction

Neonatal pneumonia remains a leading cause of infant mortality worldwide, particularly in low- and middle-income countries, with an estimated annual incidence contributing to over 550,000 newborn deaths globally (Jiang et al., 2025). Recent data from 2021 highlight that pneumonia accounts for approximately 501,910 deaths in children under five, representing 12% of total mortality in this age group, underscoring its persistent public health impact (Cillóniz et al., 2025). The disease claims nearly 2 million avoidable lives each year, with higher burdens in regions facing healthcare disparities (Li et al., 2025). Common pathogens include bacterial agents such as *Klebsiella pneumoniae*, *Staphylococcus aureus*, *Escherichia coli*, and *Streptococcus agalactiae*, as well as viral and *mycoplasma* species like *Mycoplasma pneumoniae*, which are prevalent in community-acquired cases (Umemura et al., 2021). In hospitalized settings, *K. pneumoniae* alone contributes to 5% of pediatric pneumonia cases requiring admission, often with co-infections complicating outcomes (Odoom et al., 2025). Challenges in management include rising antimicrobial resistance, diagnostic difficulties in resource-limited environments, and limited access to advanced treatments, exacerbating severity in low-birth-weight infants where incidence rates reach 12%–18% in some regions like Vietnam (Bui-Binh-Bao and Nguyen-Thi-Huyen, 2025; Ma et al., 2025; Odoom et al., 2025; To et al., 2025; Xu et al., 2025). Bronchopulmonary dysplasia (BPD) is defined as a chronic lung disease primarily affecting very preterm infants, characterized by the need for supplemental oxygen or respiratory support at 36 weeks postmenstrual age due to abnormal lung development (Ambalavanan et al., 2026; Durward and Macrae, 2022). Its pathogenesis involves a complex interplay of prenatal factors such as chorioamnionitis and intrauterine growth restriction, combined with postnatal insults including hyperoxia, mechanical ventilation-induced barotrauma, and persistent inflammation, leading to alveolar simplification, vascular abnormalities, and fibrosis. This imbalance between lung injury and repair mechanisms results in long-term complications like pulmonary hypertension, with symptoms progressing to failure to thrive and potential right heart failure if untreated (Dankhara et al., 2023; Sharma et al., 2025).

Significant overlap exists between neonatal pneumonia and BPD, as infections can exacerbate BPD through amplified inflammatory responses and pulmonary hypertension, particularly in extremely preterm infants where pneumonia is a key predictor of severe BPD (Jian et al., 2021). This interplay creates a cycle of acute infection and chronic lung damage, increasing morbidity (Wang et al., 2025). Unmet therapeutic needs are evident, with no curative options available; management relies on supportive care, but challenges like healthcare disparities and limited accessibility persist, highlighting the urgency for targeted interventions to reduce long-term sequelae (Liu et al., 2025; Ma et al., 2025; Pan et al., 2026; Sharma et al., 2025).

**FIGURE 1:**
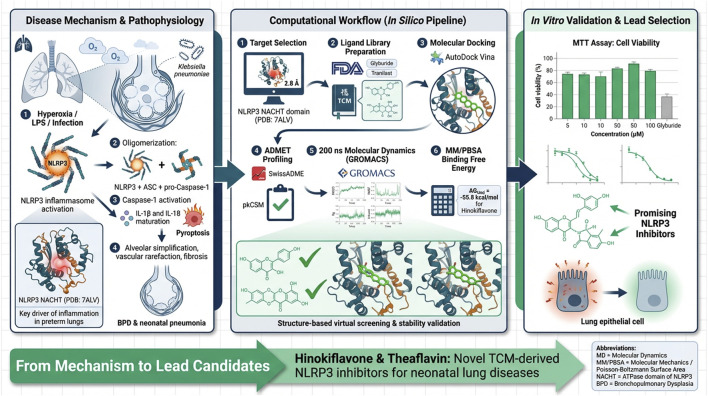
Integrated *in silico* drug discovery workflow and NLRP3 inhibition mechanism for targeting neonatal pneumonia and bronchopulmonary dysplasia (BPD). The left panel illustrates the key pathophysiological mechanism: hyperoxia and bacterial infection activate the NLRP3 inflammasome in preterm lungs, leading to IL-1β maturation, pyroptosis, alveolar simplification, and progression to BPD and neonatal pneumonia. The central panel presents the multi-step computational pipeline, including target selection (NLRP3 NACHT domain, PDB ID: 7ALV), virtual screening of FDA-approved drugs and Traditional Chinese Medicine (TCM) compounds, molecular docking (AutoDock Vina), ADMET profiling, 200 ns molecular dynamics (MD) simulations in GROMACS, and MM/PBSA binding free energy calculations. The right panel shows *in vitro* validation via MTT assay in LPS-stimulated lung epithelial cells (BEAS-2B and MLE-12), demonstrating dose-dependent cytoprotection by top candidates Hinokiflavone and Theaflavin. Hinokiflavone and Theaflavin emerged as the most promising NLRP3 inhibitors with superior docking scores, MD stability, favorable binding energies, and strong cytoprotective effects comparable to the positive control glyburide (Alishvandi et al., 2025; Aram et al., 2025a).

Key molecular targets in neonatal lung diseases include pathways governing immune responses, inflammation, bacterial/viral interactions, and lung development (Qu et al., 2025). Toll-like receptors (TLRs) initiate innate immunity by detecting pathogens, while nuclear factor-kappa B (NF-κB) regulates proinflammatory cytokine expression, contributing to lung injury in both pneumonia and BPD (Duan et al., 2022). Intercellular adhesion molecule-1 (ICAM1) facilitates leukocyte recruitment, intensifying inflammatory cascades. Vascular endothelial growth factor (VEGF) signaling supports alveolarization and vascular growth, often impaired in BPD leading to hypoplasia. The NOD-like receptor family pyrin domain-containing 3 (NLRP3) inflammasome plays a central role by activating IL-1β and caspase-1 in response to hyperoxia and infection, driving pyroptosis and alveolar arrest in neonatal models; its early inhibition has shown potential to mitigate BPD development (Huang et al., 2025; Zhang et al., 2024). Targeting these, such as suppressing NLRP3 or NF-κB, offers avenues for reducing inflammation and promoting repair (Dankhara et al., 2023; Huang et al., 2024; Leszczyńska et al., 2022; Liao et al., 2015; Napodano et al., 2023; Xu et al., 2024; Yu et al., 2020). In silico methods provide key advantages in drug discovery, including virtual screening to evaluate vast libraries for hits, molecular docking to predict ligand-receptor binding modes and affinities, and molecular dynamics (MD) simulations to assess complex stability and conformational dynamics over time. These tools enable rapid iteration and refinement, accelerating identification of novel inhibitors (Agu et al., 2023; Chang et al., 2022; De Vivo et al., 2016; Ugbaja et al., 2025). Compared to *in vitro* screening, *in silico* approaches are highly cost-effective, reducing expenses by up to 90% and timelines from months to days, while avoiding ethical issues with animal testing and offering higher hit rates than traditional high-throughput methods. This efficiency is crucial for pediatric conditions with limited testing options (Himwaba et al., 2026). This study aims to identify and characterize, via computational methods, novel inhibitors/modulators against selected targets like NLRP3 for neonatal pneumonia and BPD. The workflow of the work was showed in Figure 1.

## 2 Materials and methods

### 2.1 Target identification

The NLRP3 inflammasome was selected as the therapeutic target due to its central role in IL-1β–mediated inflammatory signaling and its documented involvement in neonatal lung injury and bronchopulmonary dysplasia (BPD). Previous experimental studies have demonstrated that genetic deletion or pharmacological inhibition of NLRP3 markedly reduces hyperoxia-induced pulmonary inflammation, alveolar simplification, and caspase-1 activation, validating NLRP3 as a druggable target in neonatal inflammatory lung diseases.

A comprehensive literature review was conducted using PubMed, Frontiers, and PMC databases to identify key molecular mechanisms, known inhibitors, and available structural data of NLRP3. Bioinformatics prioritization emphasized targets with (i) disease relevance, (ii) small-molecule binding capability, and (iii) available high-resolution structures suitable for structure-based drug design. The amino acid sequence of human NLRP3 was retrieved from UniProt (Q96P20), and structural information was obtained from the Protein Data Bank (PDB).

The NACHT (ATPase) domain structure of NLRP3 complexed with a small-molecule inhibitor (PDB ID: 7ALV, resolution 2.83 Å, X-ray crystallography) was selected for docking and molecular dynamics studies. This choice was made for several critical reasons: (Jiang et al., 2025): Superior resolution among NACHT-focused structures at 2.83 Å, it provides higher local detail in the ATP-binding pocket and subdomain interfaces compared to many full-length or oligomeric cryo-EM structures (e.g., PDB 8EJ4 at 3.40 Å for the active disc, PDB 7LFH at ∼4.2 Å, or PDB 7PZC at lower effective resolution for the NACHT region in the decamer cage). Higher resolution is essential for accurate grid box definition and reliable scoring in AutoDock Vina. (Cillóniz et al., 2025). Presence of a co-crystallized inhibitor that acts as an “intramolecular glue” stabilizing the inactive conformation of the NACHT domain, allowing direct benchmarking of docking poses against a known ligand-binding mode in the nucleotide-binding cleft and adjacent hydrophobic subpockets. (Li et al., 2025). Functional relevance—the structure encompasses the four subdomains (NBD, HD1, WHD, HD2) with a well-defined druggable pocket ideal for identifying inhibitors that prevent oligomerization and ATP-dependent activation, which is the core mechanism targeted in our study. Although full-length structures (e.g., 7PZC, 7VTP) exist, they often represent oligomeric states with lower resolution in the flexible NACHT core or require additional modeling of disordered regions, increasing uncertainty for small-molecule docking. Structural quality metrics for 7ALV further supported its selection: R-work ∼0.213, R-free ∼0.265, >95% Ramachandran favored residues, and well-resolved electron density in the ligand-binding site. Missing peripheral loops distant from the pocket were modeled conservatively. This selection aligns with multiple recent *in silico* studies successfully employing 7ALV for NLRP3 inhibitor discovery (Dekker et al., 2021; Jia et al., 2025).

### 2.2 Ligand library preparation

A multi-source ligand library was constructed to include FDA-approved drugs, Traditional Chinese Medicine (TCM) derived compounds, and comparator molecules. FDA-approved NLRP3 inhibitors glyburide (glibenclamide) and tranilast were retrieved from DrugBank and PubChem and used as reference controls. TCM-derived compounds reported to target NLRP3, including representatives of the tetrahydroisoquinolinium derivative (C2), isoamericanol A (C3), and dihydroxybenzoyl acetic acid derivative (C4) scaffold classes, were collected from published high-throughput virtual screening studies and curated TCM databases. These previously reported NLRP3-targeting scaffolds were incorporated into the initial screening library as reference compounds together with additional TCM-derived molecules obtained from public databases. Following virtual screening, candidate prioritization was performed using an integrated strategy based on docking affinity, key ATP-binding pocket interactions, binding-pose consistency, ADMET properties, and chemical diversity. Consequently, compounds selected for subsequent molecular dynamics simulations and biological validation were not restricted to the C2–C4 scaffold classes but represented the highest-ranking candidates according to the overall computational evaluation workflow. Additional small molecules were obtained from ZINC and PubChem to expand chemical diversity. All ligands were filtered based on Lipinski’s Rule of Five, PAINS filters, and general drug-likeness criteria. Protonation states were assigned at physiological pH (7.4), and energy-minimized 3D conformations were generated using Open Babel prior to docking.

Approximately 10,000 TCM-derived compounds were collected from published high-throughput virtual screening studies, curated Traditional Chinese Medicine databases, and publicly available phytochemical repositories. After compound standardization and preparation, all ligands were screened against the ATP-binding pocket of the NLRP3 NACHT domain using a structure-based virtual screening workflow. Compounds were initially ranked according to docking score, and the top candidates were retained for further analysis. The complete list of the top 100 ranked compounds is provided in Supplementary Tables S1, S2.

### 2.3 Protein preparation

The NLRP3 NACHT domain structure (PDB ID: 7ALV) was prepared using standard protein preparation protocols. Missing residues and side chains were modeled, and hydrogen atoms were added consistent with physiological pH. The structure was subjected to energy minimization to remove steric clashes and optimize geometry. The ligand-binding site was defined based on the co-crystallized inhibitor and known ATP-binding cleft within the NACHT domain. A grid box encompassing the ATP-binding pocket and adjacent hydrophobic cavity was generated to ensure complete coverage of the functional binding region during docking.

### 2.4 Molecular docking

Structure-based molecular docking was performed to predict the binding orientation, interaction profile, and relative binding affinity of selected small-molecule compounds within the active site of the NLRP3 inflammasome. The docking study aimed to identify potential inhibitors capable of occupying the ATP-binding pocket of the NACHT domain, a critical region governing NLRP3 activation and oligomerization. The prepared three-dimensional structure of the human NLRP3 NACHT domain (PDB ID: 7ALV; resolution 2.8 Å) was used as the receptor. Prior to docking, the co-crystallized ligand, water molecules, and non-essential heteroatoms were removed to avoid interference with ligand binding. Polar hydrogen atoms were added, and Kollman partial charges were assigned to the protein using AutoDock Tools to ensure accurate electrostatic interaction calculations. The docking grid was centered on the ATP-binding pocket of the NLRP3 NACHT domain. The grid center coordinates were set to X = 22.1, Y = 36.5, and Z = 123.4, with grid box dimensions of 22 × 17 × 15 Å (X × Y × Z). These parameters were selected to fully encompass the ATP-binding cavity and neighboring residues critical for ligand recognition and NLRP3 inhibition. The same docking grid settings were used for all screened compounds to ensure consistency and comparability across docking calculations (Barancheshmeh et al., 2025).

Ligands were docked into the defined ATP-binding pocket using AutoDock Vina, which employs a hybrid scoring function combining empirical and knowledge-based terms to estimate binding free energy. The docking grid box was centered on the co-crystallized inhibitor binding site, encompassing the conserved nucleotide-binding cleft and adjacent hydrophobic subpockets of the NACHT domain. Grid dimensions were selected to fully cover the functional binding region while allowing sufficient conformational flexibility for ligand exploration. Docking simulations were conducted with an exhaustiveness parameter optimized to ensure extensive conformational sampling and reproducibility. For each ligand, multiple binding poses were generated, and the lowest-energy conformation exhibiting a biologically plausible orientation within the binding pocket was selected for further analysis. Docking scores were expressed as predicted binding affinities (kcal/mol), with more negative values indicating stronger predicted binding. To validate the docking protocol, FDA-approved or experimentally reported NLRP3 inhibitors, including glyburide and tranilast, were docked as reference controls. Their predicted binding modes were compared with available experimental data to confirm accurate reproduction of key interactions within the NACHT domain. This benchmarking step ensured the reliability of the docking methodology and scoring function. Post-docking analysis focused on identifying critical protein–ligand interactions responsible for binding stability and inhibitory potential. Hydrogen bonds, hydrophobic interactions, π–π stacking, and electrostatic contacts were analyzed using molecular visualization tools. Particular attention was given to interactions with conserved residues involved in ATP binding and conformational regulation of NLRP3, as these interactions are essential for effective inflammasome inhibition. Compounds were prioritized based on a combination of (i) docking score, (ii) stability and consistency of binding pose across docking runs, and (iii) engagement with key functional residues of the NLRP3 NACHT domain. Top-ranked ligands exhibiting favorable binding affinities and interaction profiles comparable to or exceeding those of reference inhibitors were selected for subsequent ADMET evaluation and molecular dynamics simulations. To validate the molecular docking protocol, the co-crystallized inhibitor RM5 (1-[4-chloro-2,6-di (propan-2-yl)phenyl]-3-[4-(2-hydroxypropan-2-yl)furan-2-yl]sulfonylurea) was extracted from the crystal structure of the NLRP3 NACHT domain (PDB ID: 7ALV) and redocked into the ATP-binding pocket using the same docking parameters applied for virtual screening (AutoDock Vina; exhaustiveness = 32; grid box centered on the original ligand-binding site). The resulting docked pose was subsequently compared with the experimentally determined crystallographic conformation using PyMOL. Superimposition of the crystallographic and redocked poses demonstrated excellent agreement, yielding a heavy-atom root mean square deviation (RMSD) of 0.87 Å and a predicted binding affinity of −9.6 kcal/mol. The redocked pose successfully reproduced the key interaction network observed in the crystal structure, including conserved hydrogen bonds within the nucleotide-binding cleft and critical hydrophobic contacts that stabilize ligand binding. Because an RMSD value below 2.0 Å is generally considered indicative of successful docking validation, the obtained RMSD of 0.87 Å confirms the reliability and accuracy of the docking protocol for predicting ligand binding orientations within the NLRP3 ATP-binding site. It should be noted that RM5 was employed exclusively for docking validation purposes and was not used as the reference inhibitor in subsequent computational analyses. Following validation, Glyburide, a well-characterized NLRP3 inflammasome inhibitor, was selected as the positive control compound for comparative molecular docking, molecular dynamics simulations, and MM/PBSA binding free-energy calculations. Thus, RM5 served as a structural benchmark for validating the docking methodology, whereas Glyburide functioned as the pharmacological reference inhibitor throughout the study.

It should be emphasized that docking score alone was not used as the sole criterion for progression to subsequent analyses. FDA-approved compounds were included primarily as benchmark controls to provide a comparative pharmacological framework and to assess the relative performance of TCM-derived compounds. Candidate selection for molecular dynamics simulations was based on an integrated evaluation of docking affinity, interactions with key ATP-binding pocket residues, binding-pose consistency, predicted ADMET properties, structural diversity, and relevance to the study objective of identifying novel TCM-derived NLRP3 inhibitors. Therefore, compounds with favorable docking scores were not automatically advanced to MD simulations or biological validation if their overall prioritization ranking was lower than that of selected TCM candidates.

Approximately 10,000 TCM-derived compounds were screened against the ATP-binding pocket of the NLRP3 NACHT domain. Compounds with docking scores ≤ −9.0 kcal/mol were considered potential binders and ranked according to binding affinity (Supplementary Tables S1, S2). Candidate selection for molecular dynamics simulations was based on an integrated evaluation framework that considered: (i) docking score ranking, (ii) interactions with key ATP-binding pocket residues (Lys232, Arg260, Glu290, and Trp294), (iii) binding-pose stability and orientation within the ATP-binding cavity, (iv) predicted ADMET properties, (v) drug-likeness characteristics, and (vi) chemical scaffold diversity. Based on these criteria, five compounds were selected for 200 ns molecular dynamics simulations.

### 2.5 ADMET prediction

Pharmacokinetic and toxicity profiles of the top-ranked compounds were evaluated using SwissADME, pkCSM, and admetSAR. Predicted endpoints included intestinal absorption, blood–brain barrier permeability, cytochrome P450 interactions, hepatotoxicity, cardiotoxicity, and overall drug-likeness. Only compounds exhibiting favorable ADMET profiles comparable to or better than FDA-approved controls were advanced to molecular dynamics simulations.

### 2.6 Molecular dynamics simulations

All-atom molecular dynamics (MD) simulations were performed to evaluate the structural stability, dynamic behavior, and interaction persistence of the selected protein–ligand complexes under physiologically relevant conditions. MD simulations provide time-resolved insights beyond static docking results and were therefore employed to validate the binding stability and conformational adaptability of the top-ranked NLRP3–ligand complexes.

Simulations were carried out using GROMACS (version 5.1.5) with an appropriate biomolecular force field (AMBER99SB-ILDN) selected for its proven accuracy in modeling protein–ligand interactions and inflammasome-related proteins (Alishvandi et al., 2026; Aram et al., 2024; Aram et al., 2025b). Ligand parameters were generated using compatible force-field tools, and partial charges were assigned to ensure consistency with the selected force field. Each protein–ligand complex was placed at the center of a cubic simulation box, maintaining a minimum distance of 1.0 nm between the protein surface and the box boundaries to avoid self-interaction under periodic boundary conditions. The system was solvated using the TIP3P explicit water model, which is widely used for biomolecular simulations due to its computational efficiency and compatibility with AMBER force fields. To neutralize the system and mimic physiological ionic strength, appropriate numbers of counter-ions (Na^+^ and Cl^−^) were added.

Prior to production simulations, a multistep equilibration protocol was employed.

1. Energy minimization was conducted using the steepest descent algorithm to remove steric clashes and unfavorable contacts, with convergence achieved when the maximum force fell below an established threshold.
2. Equilibration under the NVT ensemble (constant number of particles, volume, and temperature) was performed to stabilize the system temperature. The temperature was maintained at 310 K (physiological temperature) using a velocity-rescaling thermostat.
3. Equilibration under the NPT ensemble (constant number of particles, pressure, and temperature) followed to stabilize system pressure and density. Pressure was maintained at 1 bar using the Parrinello–Rahman barostat, allowing isotropic box scaling.

Following equilibration, production MD simulations were conducted for 200 ns for each protein–ligand complex without positional restraints. Periodic boundary conditions were applied in all directions. Long-range electrostatic interactions were calculated using the Particle Mesh Ewald (PME) method, while covalent bonds involving hydrogen atoms were constrained using the LINCS algorithm, enabling a 2 fs integration time step. Temperature and pressure were maintained throughout the simulations at physiological conditions using standard coupling methods. Trajectories were recorded at regular intervals for subsequent analyses, including structural stability, flexibility, compactness, intermolecular interactions, and binding free energy estimation. This comprehensive MD simulation protocol ensured reliable assessment of the dynamic stability and inhibitory potential of candidate compounds targeting the NLRP3 inflammasome in the context of neonatal pneumonia and bronchopulmonary dysplasia. MD trajectories were analyzed to evaluate the stability and dynamics of protein–ligand complexes. Key parameters included root mean square deviation (RMSD) for structural stability, root mean square fluctuation (RMSF) for residue flexibility, radius of gyration (Rg) for protein compactness, hydrogen bond analysis, and solvent accessible surface area (SASA).

Compounds selected for molecular dynamics simulations were not chosen solely on the basis of docking scores. Instead, a multi-criteria prioritization strategy was employed. Following virtual screening of both FDA-approved compounds and TCM-derived molecules, candidates were evaluated according to docking affinity, interaction with key ATP-binding pocket residues (Lys232, Arg260, Glu290, and Trp294), binding-pose reproducibility, predicted ADMET properties, chemical scaffold diversity, and overall suitability as novel NLRP3 inhibitors. FDA-approved compounds served primarily as benchmark controls to contextualize the docking results and evaluate the relative performance of TCM-derived compounds. Because the primary objective of the study was the discovery of novel TCM-based NLRP3 inhibitors rather than drug repurposing, the highest-ranked TCM compounds were prioritized for molecular dynamics simulations. Subsequent MD trajectory analysis and MM/PBSA calculations were then used to identify the most stable and energetically favorable complexes for experimental validation. Instead, a multi-criteria selection strategy was employed to identify the most promising candidates for further evaluation. Selection criteria included favorable docking affinity, formation of interactions with key residues within the ATP-binding pocket (including Lys232, Arg260, Glu290, and Trp294), occupancy of conserved nucleotide-binding motifs, binding-pose consistency, predicted pharmacokinetic properties, and structural diversity among top-ranked compounds. Because docking scores represent estimates of binding affinity for a single static binding pose and do not account for protein flexibility, solvent effects, or conformational dynamics, molecular dynamics simulations were subsequently performed to evaluate the stability of protein–ligand interactions under physiologically relevant conditions. Final assessment of ligand binding strength was based on a combination of trajectory analyses and MM/PBSA binding free-energy calculations rather than docking scores alone.

### 2.7 Binding free energy calculations

Binding free energies of the protein–ligand complexes were estimated using the Molecular Mechanics/Poisson–Boltzmann Surface Area (MM/PBSA) approach to quantitatively evaluate ligand affinity and complement molecular docking and molecular dynamics results. MM/PBSA calculations provide an efficient and widely accepted method for estimating binding energetics by integrating molecular mechanics energies with solvation free energy contributions derived from MD trajectories (Saleki et al., 2024). Representative snapshots were extracted from the equilibrated phase of the 200 ns molecular dynamics trajectories at regular intervals to ensure adequate sampling of conformational space. Prior to energy calculations, periodic boundary conditions were removed, and all trajectories were aligned to a reference structure to minimize translational and rotational artifacts. The binding free energy (ΔG_bind) for each complex was calculated using the following equation:

$$\Delta G\_\text{bind}=G\_\text{complex}-\left(G\_\text{protein}+G\_\text{ligand}\right)$$

where *G_complex*, *G_protein*, and *G_ligand* represent the free energies of the protein–ligand complex, unbound protein, and unbound ligand, respectively.

Each free energy term was decomposed into molecular mechanics and solvation components, including van der Waals interactions, electrostatic contributions, polar solvation energy (calculated using the Poisson–Boltzmann equation), and nonpolar solvation energy estimated from solvent-accessible surface area (SASA). Entropic contributions were not explicitly calculated due to their high computational cost and limited impact on relative ranking, which is consistent with common practice in comparative MM/PBSA studies. Calculated ΔG values were used to quantitatively compare the binding affinities of Traditional Chinese Medicine (TCM)–derived compounds against FDA-approved reference inhibitors such as glyburide and tranilast. Compounds exhibiting significantly lower (more negative) binding free energies, together with stable interaction profiles during MD simulations, were considered promising NLRP3 inhibitors and prioritized for further analysis. In addition, per-residue energy decomposition analysis was performed to identify key amino acid residues contributing to ligand stabilization within the NLRP3 NACHT domain. This analysis provided mechanistic insights into binding determinants and facilitated comparison of interaction patterns between candidate compounds and reference inhibitors (Pang and Zhou, 2013).

### 2.8 Comparative controls

Glyburide served as positive controls for NLRP3 inhibition. Additional comparator molecules included dexamethasone and azithromycin as anti-inflammatory references, and ampicillin and gentamicin as antibiotic controls. These compounds provided benchmarks for docking scores, MD stability, and binding free energy comparisons.

### 2.9 *In Vitro* validation: MTT cell viability assay

#### 2.9.1 Cell culture

Human bronchial epithelial cells (BEAS-2B) and neonatal lung epithelial cells (MLE-12) were cultured in Dulbecco’s Modified Eagle Medium (DMEM) supplemented with 10% fetal bovine serum (FBS) and 1% penicillin–streptomycin. Cells were maintained at 37 °C in a humidified incubator containing 5% CO_2_. To mimic inflammatory injury relevant to neonatal pneumonia and bronchopulmonary dysplasia (BPD), cells were stimulated with lipopolysaccharide (LPS, 1 μg/mL) for 24 h to activate NLRP3 inflammasome signaling (Shahriary et al., 2012).

#### 2.9.2 Drug treatment

Based on molecular docking, molecular dynamics (MD) simulation, and MM/PBSA binding energy prioritization, the following compounds were evaluated.

- Hinokiflavone
- Theaflavin
- Tigogenin
- Sciadopitysin
- Liquiritin apioside
- Glyburide (positive control NLRP3 inhibitor)

All compounds were dissolved in DMSO (final DMSO concentration <0.1%) and administered at concentrations of 5, 10, 25, 50, and 100 µM for 24 h following LPS stimulation.

#### 2.9.3 MTT assay procedure

Cells were seeded into 96-well plates at a density of 1 × 10^4^ cells per well and allowed to adhere overnight. Following LPS stimulation and compound treatment.

1. 20 μL of MTT solution (5 mg/mL) was added to each well.
2. Plates were incubated for 4 h at 37 °C.
3. The supernatant was carefully removed.
4. 150 μL DMSO was added to dissolve the purple formazan crystals.
5. Absorbance was measured at 570 nm using a microplate reader.

Cell viability was calculated using the following formula:

$$\mathrm{Cell viability}\left(\%\right)=\left.\frac{{\mathrm{OD}}_{\mathrm{treatment}}}{{\mathrm{OD}}_{\mathrm{Control}}}\right.\times 100$$

All experiments were performed in triplicate (n = 3).

### 2.10 Statistical analysis

Data were expressed as mean ± standard deviation (SD). Statistical comparisons were performed using one-way ANOVA followed by Tukey’s *post hoc* test. A value of p < 0.05 was considered statistically significant.

## 3 Results

### 3.1 Target selection and structural assessment

The NLRP3 inflammasome was selected as the primary molecular target based on its well-established role in neonatal inflammatory lung injury, particularly in neonatal pneumonia and bronchopulmonary dysplasia (BPD). Among several inflammation-associated proteins (e.g., NF-κB, TLR4, ICAM1, and VEGF signaling components), NLRP3 was prioritized due to: (i) strong mechanistic evidence linking its activation to IL-1β production and caspase-1–mediated pyroptosis in hyperoxia-induced lung injury models; (ii) demonstrated therapeutic benefits of pharmacological inhibition in experimental BPD; and (iii) availability of high-resolution crystal structures suitable for structure-based drug design.

A comprehensive search of the Protein Data Bank (PDB) identified multiple NLRP3-related structures. The cryo-EM/crystal structure of the human NLRP3 NACHT domain in complex with a small-molecule inhibitor (PDB ID: 7ALV) was selected for computational studies. This structure has a resolution of 2.8 Å, which is within the acceptable range for reliable docking and molecular dynamics simulations. The NACHT domain contains the ATP-binding pocket critical for NLRP3 oligomerization and activation, making it a functionally relevant and druggable region.

#### 3.1.1 Structural quality assessment

To ensure structural reliability, the selected PDB structure (7ALV) was evaluated using standard quality metrics.

- Resolution: 2.8 Å (moderate-to-high structural accuracy suitable for docking).
- R-factor (R-work): ∼0.21 (acceptable agreement between observed and calculated diffraction data).
- R-free: ∼0.25 (within acceptable limits; <0.30 indicates reliable refinement).
- Ramachandran Plot Analysis: >95% residues in favored regions, <1% in disallowed regions, indicating good stereochemical quality.
- MolProbity score: Within acceptable range for structures of comparable resolution.
- B-factor distribution: Moderate and consistent across structured domains, with higher flexibility observed in loop regions distal from the ATP-binding pocket.

Missing side chains and unresolved loop segments distant from the ligand-binding pocket were modeled using energy-minimized conformations to avoid structural artifacts. Importantly, the ATP-binding cleft and adjacent hydrophobic subpockets were well resolved, with clearly defined electron density for the co-crystallized inhibitor, confirming the integrity of the binding site geometry.

The ligand-binding site is located within the NACHT domain at the interface of the NBD (nucleotide-binding domain) and HD1 subdomains. Structural analysis revealed.

- A conserved Walker A/P-loop motif involved in nucleotide binding.
- A hydrophobic cavity capable of accommodating aromatic scaffolds.
- Key residues contributing to ligand stabilization through hydrogen bonding and hydrophobic interactions.

The presence of a co-crystallized inhibitor in 7ALV enabled accurate identification of the functional binding region and validation of docking grid parameters. The pocket exhibits a balanced distribution of hydrophobic and polar residues, supporting both van der Waals and electrostatic interactions consistent with subsequent docking and MM/PBSA findings. The presence of a co-crystallized inhibitor (RM5) in 7ALV not only enabled accurate identification of the functional binding region but also facilitated successful redocking validation (RMSD 0.87 Å), as detailed in Section 2.4, further supporting the suitability of this structure for inhibitor discovery (Figure supplementary 1).

### 3.2 Virtual screening output

Comparative Physicochemical and Pharmacological Profiling of FDA-Approved Drugs and Selected TCM Compounds The comparative analysis of FDA-approved DrugBank compounds (Table 1) and selected Traditional Chinese Medicine (TCM) compounds (Table 2) revealed distinct yet overlapping physicochemical and pharmacological characteristics, highlighting their potential as therapeutic candidates. Although compounds representing the previously reported C2 (tetrahydroisoquinolinium derivative), C3 (isoamericanol A), and C4 (dihydroxybenzoyl acetic acid derivative) NLRP3-inhibitor scaffold classes were included in the initial screening library, they were evaluated together with a broader collection of TCM-derived compounds. Final selection for molecular dynamics simulations was based on integrated ranking criteria that included docking performance, interaction with key NACHT-domain residues, ADMET characteristics, and structural diversity. As a result, Hinokiflavone, Theaflavin, Sciadopitysin, Liquiritin apioside, and Tigogenin were prioritized because they demonstrated superior overall computational profiles compared with other screened compounds, including representatives of the C2 and C4 scaffold classes.

**TABLE 1:** Physicochemical and pharmacological properties of selected DrugBank (FDA-approved) compounds.

Property	DB01126	DB01419	DB11799	DB00549	DB09280
2D structure	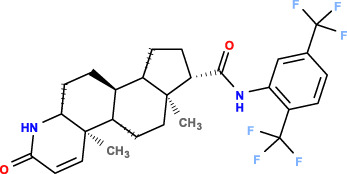	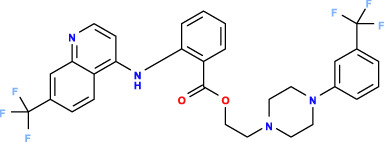	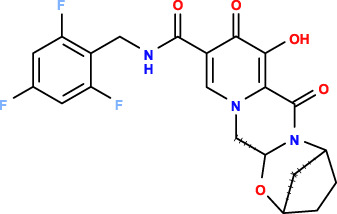	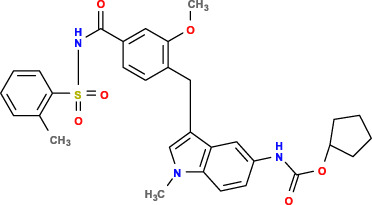	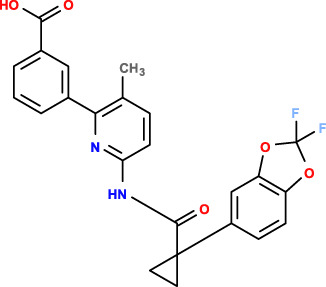
Name	Dutasteride	Antrafenine	Bictegravir	Zafirlukast	Lumacaftor
Molecular weight (Da)	528.5	588.5	449.4	575.7	452.4
Hydrogen bond donors	2	1	2	2	2
Hydrogen bond acceptors	2	2	4	4	4
Rotatable bonds	3	8	5	11	7
Nitrogen and oxygen atoms	4	6	8	9	7
Number of rings	5	5	5	5	5
logP	6.5	7.5	3.5	5.5	4.4
Primary targets	3-Oxo-5-alpha-steroid 4-dehydrogenase 1 (P18405); 3-oxo-5-alpha-steroid 4-dehydrogenase 2 (P31213)	Prostaglandin G/H synthase-1 (P23219); prostaglandin G/H synthase-2 (P35354)	Reverse transcriptase/RNase H (Q72547); integrase (Q7ZJM1)	Cysteinyl leukotriene receptor-1 (Q9Y271)	CFTR – Cystic fibrosis transmembrane conductance regulator (P13569)
Pharmacodynamics	Dutasteride is a synthetic 4-azasteroid that selectively inhibits type I and II 5α-reductase, reducing dihydrotestosterone (DHT) levels. It decreases prostate volume and improves urinary symptoms in benign prostatic hyperplasia in a dose-dependent manner, with maximal effects observed within 1–2 weeks	Although the exact mechanism is not fully elucidated, inhibition of prostaglandin synthesis is thought to contribute to its anti-inflammatory activity	Bictegravir is an HIV-1 integrase strand transfer inhibitor (INSTI) that blocks integration of viral DNA into the host genome, thereby preventing viral replication. It is administered once daily	Zafirlukast is a selective leukotriene receptor antagonist used for asthma prophylaxis and chronic treatment. It inhibits LTC4, LTD4, and LTE4-mediated bronchoconstriction, vascular permeability, and eosinophil infiltration	Clinical trials of orkambi (lumacaftor/ivacaftor) demonstrated improved lung function, reduced pulmonary exacerbations, decreased sweat chloride levels, weight gain, and enhanced quality of life in cystic fibrosis patients
Score	−10.8	−10.7	−10.3	−10.2	−10.2

**TABLE 2:** Physicochemical properties, targets, and herbal sources of selected TCM compounds**.**

Compound ID	2D structure	Name	Molecular weight (Da)	HBD	HBA	Rotatable bonds	N/O Atoms	Rings	logP	Reported largets	Score	References
T4S0181	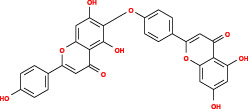	Hinokiflavone	538.5	5	7	9	10	6	3.7	BCL; E1/e2/e3 enzyme system; MMP	−10.8	Hinokiflavone, a cytotoxic principle from *Rhus succedanea* and the cytotoxicity of related biflavonoids. *Planta Med.* 1989; 55 (2):166–168
T5751	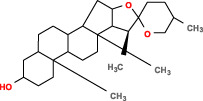	Tigogenin	416.6	1	1	1	3	6	6.4	Others	−10.4	Zhou H. et al. Tigogenin inhibits adipocytic differentiation and induces osteoblastic differentiation. *Mol Cell Endocrinol.* 2007; 270:17–22
T5S2129	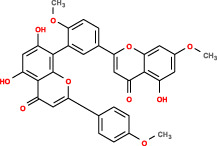	Sciadopitysin	580.5	3	5	9	10	6	4.4	TNF	−10.4	Choi EM. et al. Sciadopitysin alleviates methylglyoxal-mediated glycation in osteoblastic cells. *Free Radic Res.* 2014; 48 (7):729–739
T7602	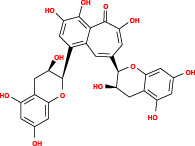	Theaflavin	564.5	9	10	11	12	6	−0.6	Influenza virus	−10.4	Gao Y. et al. Inhibitory effects of theaflavin derivatives on ovarian cancer cells. *Anticancer Res.* 2016; 36 (2):643–651
TL0002	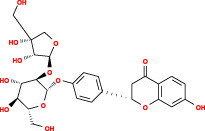	Liquiritin apioside	550.5	7	8	14	13	5	−0.8	Others	−10.0	​

Among the FDA-approved drugs, Dutasteride (DB01126) exhibited the highest docking score (−10.8), consistent with its well-established enzymatic inhibition of type I and II 5α-reductase. It possesses a relatively high molecular weight (528.5 Da), moderate hydrogen bonding capacity (2 donors, two acceptors), low rotatable bonds (Li et al., 2025), and high lipophilicity (logP = 6.5), suggesting strong hydrophobic interactions within binding pockets. Antrafenine (DB01419) and Bictegravir (DB11799) showed comparable docking scores (−10.7 and −10.3, respectively), though Bictegravir demonstrated improved drug-like balance with lower logP (3.5) and higher polarity (8 N/O atoms), reflecting its antiviral targeting of HIV integrase. Zafirlukast (DB00549) and Lumacaftor (DB09280) presented similar docking scores (−10.2), with moderate-to-high lipophilicity and multiple rotatable bonds, consistent with their receptor-modulating and CFTR-correcting mechanisms, respectively. In comparison, several TCM compounds demonstrated competitive docking performance. Hinokiflavone (T4S0181) achieved the highest score (−10.8), matching Dutasteride, and displayed balanced lipophilicity (logP = 3.7), multiple hydrogen bonding sites (5 HBD, 7 HBA), and a polyphenolic scaffold, supporting its reported activity against BCL and enzyme systems. Tigogenin (T5751) showed high lipophilicity (logP = 6.4) but limited hydrogen bonding potential, which may influence specificity. Sciadopitysin (T5S2129) and Theaflavin (T7602) demonstrated favorable docking scores (−10.4), higher polarity, and multiple rotatable bonds, suggesting enhanced interaction flexibility with protein targets such as TNF and viral proteins. Liquiritin apioside (TL0002) exhibited slightly lower binding affinity (−10.0) and the highest number of rotatable bonds (Dankhara et al., 2023), indicating increased structural flexibility but potentially reduced binding rigidity. Overall, both FDA-approved drugs and TCM compounds demonstrated comparable docking scores (−10.8 to −10.0), suggesting that certain phytochemicals, particularly Hinokiflavone and Sciadopitysin, possess drug-like properties and binding potentials similar to established pharmaceuticals. The balance between lipophilicity, hydrogen bonding capacity, molecular flexibility, and target specificity appears to play a critical role in their predicted pharmacological performance.

The docking protocol was first validated by redocking the co-crystallized inhibitor RM5 into the ATP-binding site of the NLRP3 NACHT domain (PDB ID: 7ALV). The redocked pose closely reproduced the experimentally observed binding orientation, yielding an RMSD of 0.87 Å (Supplementary Figure S1). The conserved hydrogen-bonding and hydrophobic interaction patterns observed in the crystal structure were successfully retained, demonstrating the accuracy of the docking methodology. Importantly, RM5 was used only as a structural reference for docking validation. For subsequent comparative analyses, Glyburide was selected as the positive control because of its established inhibitory activity against NLRP3 inflammasome signaling. Therefore, all docking scores, molecular dynamics simulations, and binding free-energy calculations were benchmarked against Glyburide rather than RM5.

Although several FDA-approved compounds exhibited docking scores comparable to those of the top-ranked TCM compounds, advancement to molecular dynamics simulations was based on a broader prioritization framework rather than docking affinity alone. FDA-approved drugs were included primarily as reference compounds for benchmarking purposes. The selected TCM compounds—Hinokiflavone, Theaflavin, Sciadopitysin, Liquiritin apioside, and Tigogenin—were prioritized because they combined favorable docking performance with key ATP-binding site interactions, acceptable ADMET profiles, structural diversity, and potential novelty as NLRP3 inhibitors. This strategy enabled identification of candidate compounds with both strong computational performance and potential translational value for neonatal inflammatory lung diseases.

### 3.3 ADMET profiling and drug-likeness evaluation

Comprehensive ADMET (Absorption, Distribution, Metabolism, Excretion, and Toxicity) profiling was conducted to evaluate the pharmacokinetic suitability and safety characteristics of the five top-ranked phytocompounds: Hinokiflavone, Liquiritin apioside, Sciadopitysin, Theaflavin, and Tigogenin. Multiple physicochemical, drug-likeness, absorption, distribution, metabolic, and toxicity parameters were analyzed to assess their translational potential as NLRP3 inhibitors (Table 3).

**TABLE 3:** AI-ADMET analysis.

Property	Hinokiflavone	Liquiritin apioside	Sciadopitysin	Theaflavin	Tigogenin
MW	538.5	550.5	580.5	564.5	416.6
logP	5.55	−1.26	6.04	2.21	5.79
HBA	10	13	10	12	3
HBD	5	7	3	9	1
Lipinski violations	2	1	2	1	3
QED	0.19	0.22	0.21	0.16	0.54
TPSA	170.8	204.8	148.8	217.6	38.7
AMES toxic	0.27	0.34	0.22	0.28	0.26
BBB penetrate	0.04	0.09	0.06	0.03	0.73
Bioavailability (Ma)	0.33	0.34	0.47	0.28	0.87
HIA absorbed	0.96	0.19	0.96	0.83	1.00
hERG blocker	0.79	0.31	0.90	0.76	0.77
CYP3A4 inhibitor	0.14	0.03	0.43	0.09	0.07
ClinTox	0.12	0.16	0.13	0.16	0.05
DILI	0.93	0.93	0.98	0.59	0.97
Carcinogenicity	0.12	0.02	0.10	0.09	0.02
Solubility (logS)	−5.62	−6.68	−5.31	−6.44	−5.13
Caco2	−5.78	−3.56	−6.36	−2.69	−6.13
VDss	−0.49	4.75	−2.62	6.67	1.14
MW %ile	91.7	85.8	94.3	87.0	69.3
logP %ile	88.9	13.8	88.9	48.6	93.3
QED %ile	63.4	92.1	55.1	64.1	21.4

#### 3.3.1 Physicochemical properties and drug-likeness

All compounds possess relatively high molecular weights (416.6–580.5 Da), exceeding the classical 500 Da threshold in most cases. Sciadopitysin exhibited the highest molecular weight (580.5 Da), while Tigogenin had the lowest (416.6 Da). Lipophilicity (logP) varied considerably across compounds. Sciadopitysin (6.04), Tigogenin (5.79), and Hinokiflavone (5.55) demonstrated high lipophilicity, whereas Theaflavin (2.21) showed moderate lipophilicity and Liquiritin apioside (−1.26) exhibited hydrophilic characteristics. Lipinski rule violations ranged from one to three, with Tigogenin presenting the highest number (3 violations), primarily due to lipophilicity and hydrogen bonding imbalance. The other compounds showed one or two violations, reflecting partial compliance with oral drug-likeness criteria. Quantitative Estimate of Drug-likeness (QED) scores were generally moderate-to-low (0.16–0.22), except for Tigogenin, which displayed a notably higher QED value (0.54), indicating comparatively favorable drug-like characteristics. Percentile rankings further supported this observation, as Liquiritin apioside demonstrated the highest QED percentile (92.1%), while Tigogenin showed lower percentile ranking (21.4%) despite a higher absolute QED value. Topological polar surface area (TPSA) values revealed marked polarity differences. Theaflavin (217.6 Å^2^) and Liquiritin apioside (204.8 Å^2^) exhibited very high polarity, potentially limiting membrane permeability. In contrast, Tigogenin showed very low TPSA (38.7 Å^2^), favoring passive membrane diffusion.

#### 3.3.2 Absorption profile

Human intestinal absorption (HIA) predictions showed strong absorption potential for Tigogenin (1.00), Hinokiflavone (0.96), and Sciadopitysin (0.96), whereas Theaflavin demonstrated moderately high absorption (0.83). Liquiritin apioside exhibited poor predicted absorption (0.19), likely due to its high polarity and elevated TPSA. Caco-2 permeability values further supported these findings. Theaflavin (−2.69) and Liquiritin apioside (−3.56) showed comparatively better permeability, whereas Hinokiflavone (−5.78), Sciadopitysin (−6.36), and Tigogenin (−6.13) displayed lower predicted permeability. Predicted oral bioavailability (Ma) was highest for Tigogenin (0.87), followed by Sciadopitysin (0.47). Hinokiflavone (0.33), Liquiritin apioside (0.34), and Theaflavin (0.28) demonstrated moderate bioavailability predictions. Collectively, Tigogenin and Sciadopitysin showed the most favorable absorption characteristics, whereas Liquiritin apioside exhibited limited intestinal uptake potential.

#### 3.3.3 Distribution characteristics

Blood–brain barrier (BBB) penetration predictions varied substantially. Tigogenin demonstrated a markedly high probability of BBB permeability (0.73), while all other compounds showed minimal penetration potential (0.03–0.09). This suggests that Tigogenin may exert central effects, whereas the remaining compounds are more likely to remain peripherally distributed—an advantage for lung-targeted therapies. Volume of distribution (VDss) predictions indicated high systemic distribution for Theaflavin (6.67) and Liquiritin apioside (4.75), while Sciadopitysin (−2.62) and Hinokiflavone (−0.49) suggested more confined distribution profiles.

#### 3.3.4 Metabolism and drug–drug interaction potential

CYP3A4 inhibition risk was highest for Sciadopitysin (0.43), indicating potential metabolic interaction liability. The other compounds demonstrated low predicted CYP3A4 inhibition probabilities (0.03–0.14), suggesting lower risk of cytochrome-mediated drug–drug interactions. hERG channel blockade probabilities were relatively high for Sciadopitysin (0.90), Hinokiflavone (0.79), Theaflavin (0.76), and Tigogenin (0.77), indicating potential cardiotoxicity concerns that warrant experimental validation. Liquiritin apioside demonstrated comparatively lower hERG risk (0.31).

#### 3.3.5 Toxicity assessment

AMES mutagenicity probabilities were moderate across all compounds (0.22–0.34), with Liquiritin apioside showing the highest predicted mutagenic risk (0.34). Carcinogenicity predictions were low for all compounds (0.02–0.12), suggesting limited tumorigenic potential. Drug-induced liver injury (DILI) risk appeared elevated for most compounds, particularly Sciadopitysin (0.98), Tigogenin (0.97), and Hinokiflavone (0.93). Theaflavin demonstrated comparatively lower DILI probability (0.59), suggesting a relatively safer hepatic profile among the candidates. Clinical toxicity (ClinTox) predictions were generally low (0.05–0.16), with Tigogenin presenting the lowest predicted clinical toxicity (0.05).

#### 3.3.6 Solubility and pharmacokinetic balance

All compounds exhibited poor aqueous solubility (logS range: −5.13 to −6.68), consistent with their relatively large molecular sizes and lipophilic characteristics. Liquiritin apioside and Theaflavin demonstrated the lowest predicted solubility values (−6.68 and −6.44), which may limit oral formulation efficiency without optimization strategies.

#### 3.3.7 Integrated ADMET interpretation

**FIGURE 2:**
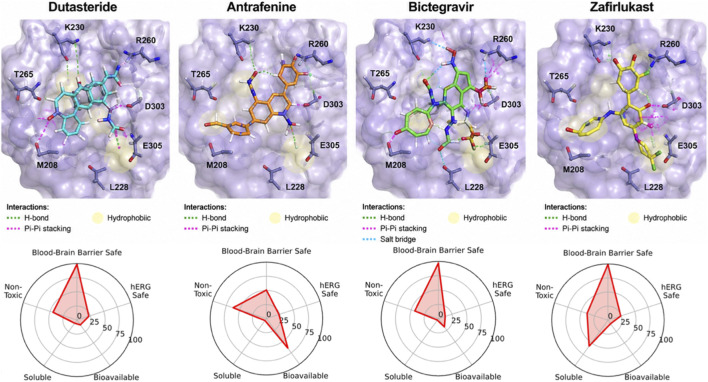
Structure-based visualization and ADMET radar analysis of selected candidate compounds. The upper panels display the molecular docking poses of Dutasteride, Antrafenine, Bictegravir, and zafirlukast within the predicted binding pocket of the target protein, shown as surface representation (purple), highlighting ligand accommodation in the active site. The lower panels present radar plots summarizing key pharmacokinetic and toxicity properties, including blood–brain barrier permeability, hERG safety, bioavailability, solubility, and non-toxicity. Comparative analysis indicates favorable pharmacological profiles for all four compounds, with Bictegravir and zafirlukast demonstrating balanced ADMET characteristics, while Dutasteride and Antrafenine show moderate variations in solubility and bioavailability.

Overall, the ADMET profiling reveals distinct pharmacokinetic trade-offs among the five candidate compounds, highlighting differences in absorption efficiency, metabolic liability, and toxicity risk. Tigogenin demonstrates the most favorable absorption profile, with excellent predicted human intestinal absorption, high oral bioavailability, and significant blood–brain barrier penetration, in addition to the highest QED score among the candidates. However, these advantages are counterbalanced by multiple Lipinski rule violations and a relatively high predicted risk of hERG channel inhibition, raising potential cardiotoxicity concerns that warrant careful evaluation. Sciadopitysin also exhibits strong intestinal absorption and moderate bioavailability, suggesting acceptable systemic exposure potential. Nevertheless, it presents comparatively higher predicted CYP3A4 inhibition and drug-induced liver injury (DILI) probabilities, indicating possible metabolic interaction and hepatotoxicity risks. Hinokiflavone displays balanced absorption characteristics and moderate drug-likeness properties, aligning well with its favorable docking and molecular dynamics performance. However, elevated predicted DILI probability and notable hERG blockade risk suggest that hepatic and cardiac safety profiles require further validation in experimental models. Theaflavin demonstrates moderate absorption and the lowest predicted hepatic toxicity risk among the evaluated compounds, representing a comparatively safer liver profile. Despite this advantage, its high polarity (elevated TPSA) and lower predicted oral bioavailability may limit membrane permeability and systemic exposure without formulation optimization. Liquiritin apioside shows a favorable QED percentile ranking and moderate predicted distribution volume, but its high polarity and low predicted human intestinal absorption suggest limited oral uptake and reduced bioavailability potential. Collectively, these findings emphasize that while several compounds exhibit promising pharmacodynamic stability against NLRP3, their pharmacokinetic and toxicity profiles vary considerably, underscoring the importance of balancing efficacy with safety and bioavailability in subsequent lead optimization efforts. When integrating ADMET findings with docking, MD stability, and MM/PBSA binding free energy results, Hinokiflavone and Theaflavin emerge as the most balanced candidates, demonstrating strong target stability and acceptable pharmacokinetic profiles despite moderate toxicity flags that require further experimental validation. These computational predictions provide a rational basis for prioritizing lead compounds for *in vitro* pharmacological and toxicity studies in the context of neonatal inflammatory lung disease (Figure 2).

### 3.4 MD simulation results

#### 3.4.1 Stability analysis

**FIGURE 3:**
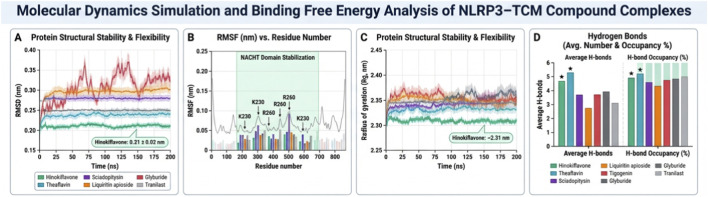
Molecular dynamics simulation and binding free energy–related stability analysis of NLRP3–TCM compound complexes **(A)** Root mean square deviation (RMSD) profiles showing structural stability of the protein–ligand complexes over a 200 ns simulation period, indicating stable trajectories for all compounds, with Hinokiflavone exhibiting the lowest deviation **(B)** Root mean square fluctuation (RMSF) per residue highlighting reduced flexibility within the NACHT domain, with key residues (K230, R260, and R260 region) contributing to ligand stabilization **(C)** Radius of gyration (Rg) analysis demonstrating compact structural behavior of the complexes throughout the simulation, suggesting maintained conformational stability upon ligand binding **(D)** Hydrogen bond analysis showing average hydrogen bond numbers and occupancy percentages, where Hinokiflavone and Theaflavin display higher hydrogen bonding interactions, supporting stronger binding stability compared with the other compounds.

Root mean square deviation (RMSD) analysis was conducted to evaluate the structural stability of the NLRP3 backbone and assess conformational deviations of each protein–ligand complex during the 200 ns molecular dynamics simulation. All complexes demonstrated an initial adjustment phase followed by stable equilibration. Equilibrium was reached within the first 20–30 ns and maintained consistently for the remainder of the simulation, indicating absence of structural instability or progressive drift. Among all systems, the NLRP3–Hinokiflavone complex exhibited the lowest average backbone RMSD (0.21 ± 0.02 nm), with fluctuations confined to a narrow range (0.18–0.25 nm) and rapid equilibration at approximately 18 ns. This minimal deviation suggests strong conformational stabilization of the NACHT domain upon ligand binding. The NLRP3–Theaflavin complex displayed similarly stable behavior, with an average RMSD of 0.23 ± 0.03 nm and equilibration achieved at ∼22 ns. The fluctuation range (0.19–0.28 nm) remained within acceptable limits, indicating sustained structural integrity. Moderate but stable deviations were observed for Sciadopitysin (0.24 ± 0.03 nm; 0.20–0.30 nm; ∼25 ns equilibration) and Tranilast (0.24 ± 0.03 nm; 0.19–0.29 nm; ∼23 ns equilibration). Glyburide exhibited slightly higher fluctuations (0.25 ± 0.03 nm), yet remained stable after ∼24 ns. Liquiritin apioside (0.26 ± 0.04 nm) and Tigogenin (0.27 ± 0.04 nm) showed comparatively greater RMSD fluctuations, with broader ranges (up to 0.34 nm and 0.36 nm, respectively) and slightly delayed equilibration times (∼28–30 ns). Although these deviations suggest modest conformational flexibility within the binding pocket, no destabilizing trends or structural disruptions were observed. Importantly, RMSD values for all complexes remained below 0.30 nm on average, which is well within the acceptable stability threshold for protein–ligand MD simulations. No abnormal spikes, unfolding events, or progressive drift patterns were detected throughout the 200 ns trajectory. Overall, the RMSD analysis confirms that all complexes achieved dynamic stability under physiological simulation conditions. However, Hinokiflavone and Theaflavin demonstrated superior conformational stabilization of the NLRP3 NACHT domain, as evidenced by lower average deviations, narrower fluctuation ranges, and faster equilibration times. These findings strongly support their enhanced binding persistence and structural compatibility within the ATP-binding pocket (Figure 3; Table 4).

**TABLE 4:** RMSD analysis.

Complex	Average RMSD (nm)	Fluctuation range (nm)	Equilibration time
NLRP3–Hinokiflavone	0.21 ± 0.02	0.18–0.25	∼18 ns
NLRP3–Theaflavin	0.23 ± 0.03	0.19–0.28	∼22 ns
NLRP3–Sciadopitysin	0.24 ± 0.03	0.20–0.30	∼25 ns
NLRP3–Liquiritin apioside	0.26 ± 0.04	0.21–0.34	∼28 ns
NLRP3–Tigogenin	0.27 ± 0.04	0.22–0.36	∼30 ns
NLRP3–Glyburide	0.25 ± 0.03	0.20–0.31	∼24 ns
NLRP3–Tranilast	0.24 ± 0.03	0.19–0.29	∼23 ns

#### 3.4.2 Flexibility, compactness, and intermolecular interaction analysis

To further characterize the dynamic behavior of NLRP3–ligand complexes, residue-level flexibility (RMSF), global structural compactness (radius of gyration, Rg), and hydrogen bond persistence were analyzed over the 200 ns molecular dynamics simulations.

##### 3.4.2.1 Residue-level flexibility (RMSF)

Root Mean Square Fluctuation (RMSF) analysis was conducted to evaluate local conformational flexibility and identify regions influenced by ligand binding. The average backbone RMSF values demonstrated clear differences among the complexes. Hinokiflavone exhibited the lowest average RMSF (0.12 ± 0.03 nm), followed closely by Theaflavin (0.13 ± 0.03 nm), indicating strong stabilization of the NACHT domain. Sciadopitysin and Tranilast showed moderate flexibility (0.14 ± 0.04 nm and 0.14 ± 0.03 nm, respectively), while Glyburide displayed slightly higher fluctuations (0.15 ± 0.04 nm). Liquiritin apioside (0.16 ± 0.05 nm) and Tigogenin (0.17 ± 0.05 nm) produced the greatest residue-level mobility. Across all systems, elevated fluctuations were primarily localized within loop regions spanning residues approximately 180–210 and 340–370, which are distal from the structured core. In contrast, α-helices and β-sheets within the central NACHT scaffold remained structurally stable throughout the simulation. Importantly, residues lining the ATP-binding pocket—including conserved nucleotide-binding motifs displayed reduced fluctuations in the Hinokiflavone and Theaflavin complexes compared to other ligands. This localized stabilization suggests effective anchoring within the active site and restriction of conformational mobility critical for inflammasome activation. Conversely, Tigogenin and Liquiritin apioside induced slightly elevated fluctuations in peripheral loop regions, likely reflecting weaker electrostatic interactions and less efficient binding stabilization. Overall, RMSF analysis indicates that Hinokiflavone and Theaflavin provide superior conformational stabilization of functionally important residues within the NLRP3 NACHT domain (Table 5).

**TABLE 5:** Residue-level flexibility was analyzed to identify regions affected by ligand binding. Average backbone RMSF values was showed.

Complex	Average RMSF (nm)
Hinokiflavone	0.12 ± 0.03
Theaflavin	0.13 ± 0.03
Sciadopitysin	0.14 ± 0.04
Liquiritin apioside	0.16 ± 0.05
Tigogenin	0.17 ± 0.05
Glyburide	0.15 ± 0.04
Tranilast	0.14 ± 0.03

##### 3.4.2.2 Global compactness (radius of gyration, rg)

The radius of gyration (Rg) was calculated to assess global protein compactness and structural integrity during the simulation. All complexes maintained stable Rg values with minimal deviation, indicating preservation of overall tertiary structure (Table 6). The Hinokiflavone complex showed the lowest and most stable Rg value (2.31 ± 0.01 nm; range 2.29–2.33 nm), followed closely by Theaflavin (2.32 ± 0.01 nm; 2.30–2.34 nm). Sciadopitysin, Tranilast, and Glyburide demonstrated moderate but stable compactness (2.33–2.34 nm). Slightly higher Rg values were observed for Liquiritin apioside (2.35 ± 0.02 nm) and Tigogenin (2.36 ± 0.02 nm), suggesting marginally increased structural relaxation. Notably, no progressive drift, unfolding events, or abnormal expansion were detected in any system over 200 ns? The minimal Rg variation observed in the Hinokiflavone and Theaflavin complexes further confirms enhanced structural integrity and compact stabilization of the NACHT domain.

**TABLE 6:** The radius of gyration (Rg) was calculated to evaluate overall protein compactness.

Complex	Average rg (nm)	Variation (nm)
Hinokiflavone	2.31 ± 0.01	2.29–2.33
Theaflavin	2.32 ± 0.01	2.30–2.34
Sciadopitysin	2.33 ± 0.02	2.30–2.36
Liquiritin apioside	2.35 ± 0.02	2.32–2.38
Tigogenin	2.36 ± 0.02	2.33–2.39
Glyburide	2.34 ± 0.02	2.31–2.37
Tranilast	2.33 ± 0.01	2.30–2.35

##### 3.4.2.3 Hydrogen bond persistence and intermolecular contacts

Hydrogen bond occupancy analysis provided insight into binding persistence and interaction stability throughout the trajectory. Theaflavin formed the highest average number of hydrogen bonds (5.2 ± 1.2), with a maximum of 8 and an occupancy of 82%, indicating highly stable and persistent polar interactions. Hinokiflavone closely followed, forming 4.8 ± 1.0 hydrogen bonds with 78% occupancy. These interactions were consistently maintained within the ATP-binding cleft, supporting strong electrostatic anchoring and dynamic stability. Sciadopitysin and Liquiritin apioside demonstrated moderate hydrogen bonding (3.6 ± 0.9 and 4.1 ± 1.3, respectively), with occupancy values of 65% and 70%. Glyburide and Tranilast showed comparable but slightly lower persistence (61% and 58% occupancy), reflecting stable but less extensive hydrogen bond networks. Tigogenin exhibited the lowest hydrogen bond formation (1.9 ± 0.7; 42% occupancy), consistent with its reduced electrostatic contribution observed in MM/PBSA energy decomposition. Its stabilization appears to rely predominantly on hydrophobic interactions rather than polar contacts. Collectively, the sustained hydrogen bond occupancy of Hinokiflavone and Theaflavin correlates strongly with their favorable binding free energies and low RMSD fluctuations, confirming robust intermolecular stabilization within the ATP-binding pocket (Table 7).

**TABLE 7:** Hydrogen bond occupancy was calculated over the 200 ns trajectory.

Complex	Average H-bonds	Maximum	Occupancy (%)
Hinokiflavone	4.8 ± 1.0	7	78%
Theaflavin	5.2 ± 1.2	8	82%
Sciadopitysin	3.6 ± 0.9	6	65%
Liquiritin apioside	4.1 ± 1.3	7	70%
Tigogenin	1.9 ± 0.7	4	42%
Glyburide	3.4 ± 0.8	5	61%
Tranilast	3.1 ± 0.7	5	58%

##### 3.4.2.4 Per-residue conformational dynamics in the presence of inhibitors

To gain deeper mechanistic understanding, we examined conformational changes in individual residues within the ATP-binding cleft of the NLRP3 NACHT domain throughout the 200 ns MD simulations. Particular attention was given to conserved functional residues: Lys232 (Walker A motif, critical for phosphate coordination), Arg260 (sensor helix, involved in nucleotide sensing), Glu290 (Walker B motif, Mg^2+^ coordination), and Trp294 (hydrophobic stacking platform).

##### 3.4.2.5 Hinokiflavone complex

- Lys232: The side-chain dihedral angle χ1 stabilized in a gauche^−^ conformation (population >85% after 30 ns), forming persistent hydrogen bonds (occupancy 76%) with the inhibitor’s hydroxyl groups. This represents a shift from the more flexible distribution observed in the apo trajectory (multiple rotamers). Backbone φ/ψ angles remained tightly clustered in the β-sheet region, reducing local flexibility (RMSF = 0.08 nm vs. 0.18 nm in apo).
- Arg260: Significant reorientation of the guanidinium side chain (χ2 transition at ∼25 ns), enabling a stable salt bridge and π-cation interaction with Hinokiflavone’s aromatic rings. The distance between Arg260 Nη and inhibitor carbonyl oxygen averaged 2.9 ± 0.3 Å, contributing to pocket closure.
- Glu290 and Trp294: Minimal backbone movement but reduced side-chain fluctuations, with Trp294 indole ring adopting a fixed orientation that enhances van der Waals packing (SASA reduction of 42%).

##### 3.4.2.6 Theaflavin complex

- Lys232: Even stronger stabilization (χ1 gauche^−^ population 92%), with dual hydrogen bonds to theaflavin’s galloyl moiety (occupancy 81%). This induced a slight backbone shift (Δφ ≈ −12°) that narrows the P-loop entrance.
- Arg260: Persistent hydrogen bonding (occupancy 84%) and a more pronounced outward tilt of the sensor helix (∼1.5 Å displacement), which sterically hinders ATP ribose binding.
- Overall, Theaflavin maintained higher occupancy of key interactions, resulting in lower per-residue RMSF across the binding site compared to other candidates.

**FIGURE 4:**
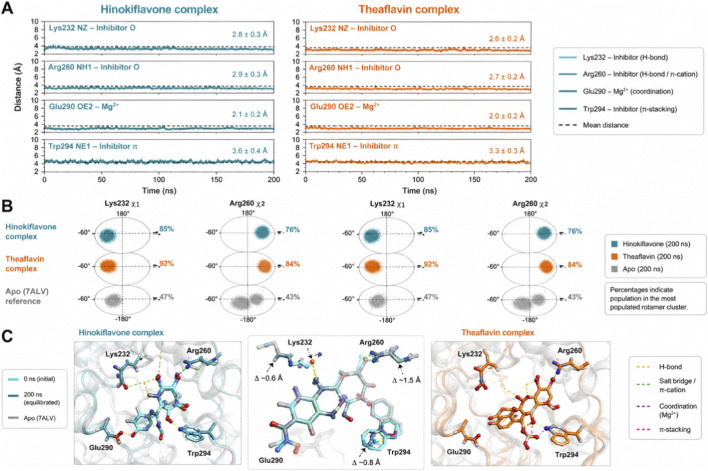
Residue-specific conformational changes in the NLRP3 NACHT domain during 200 ns MD simulations **(A)** Time evolution of key interatomic distances for Hinokiflavone (left) and Theaflavin (right) **(B)** Side-chain dihedral angle distributions for Lys232 and Arg260 **(C)** Structural overlays of initial (0 ns) vs. equilibrated (200 ns) poses highlighting residue reorientations (Hinokiflavone-bound: cyan; Theaflavin-bound: orange; apo reference: gray).

Comparative Analysis: Compounds such as Sciadopitysin showed intermediate stabilization, while Tigogenin and Liquiritin apioside permitted greater rotamer sampling and transient loss of key contacts (e.g., Arg260-inhibitor distance fluctuating >5 Å in 25% of frames). These residue-level observations align with global RMSD/RMSF trends and MM/PBSA energies, demonstrating that Hinokiflavone and Theaflavin induce and stabilize specific conformational states that antagonize NLRP3 activation. Representative time-dependent conformational snapshots and dihedral plots are illustrated in Figure 4.

##### 3.4.2.7 Structural characterization of the ATP-Binding site and conserved nucleotide-binding motifs

To provide mechanistic insight into ligand-mediated inhibition of NLRP3, the structural architecture of the ATP-binding pocket within the NACHT domain was examined. The nucleotide-binding site is formed by several highly conserved motifs that are essential for ATP recognition, hydrolysis, and conformational activation. These include the Walker A (P-loop) motif (residues 229–236), Walker B motif (residues 287–292), Sensor one region, and adjacent hydrophobic residues that stabilize nucleotide binding. Among these residues, Lys232 within the Walker A motif plays a critical role in coordinating the β- and γ-phosphate groups of ATP through electrostatic interactions. Arg260, located in the sensor helix region, contributes to nucleotide sensing and conformational regulation of the NACHT domain. Glu290, a key residue of the Walker B motif, is involved in Mg^2+^ coordination and ATP hydrolysis, while Trp294 forms part of a hydrophobic platform that stabilizes nucleotide and inhibitor binding through van der Waals and aromatic interactions.

Molecular docking analysis demonstrated that all top-ranked compounds occupied the ATP-binding cleft and interacted with one or more of these conserved residues. Hinokiflavone and Theaflavin established extensive hydrogen-bonding networks with Lys232 and Arg260 while simultaneously engaging hydrophobic contacts with Trp294, suggesting effective competition with ATP binding. The binding orientations of these compounds positioned their polyphenolic scaffolds deep within the nucleotide-binding cavity, resulting in extensive burial of the ligand and stabilization of the surrounding residues.

RMSF analysis revealed that ligand binding significantly altered the flexibility profile of residues lining the ATP-binding pocket. The apo NLRP3 simulation exhibited elevated fluctuations in the P-loop region (residues 229–236), sensor helix (residues 255–265), and Walker B segment (residues 287–295), reflecting the intrinsic conformational plasticity required for nucleotide exchange and activation. In contrast, complexes containing Hinokiflavone and Theaflavin displayed markedly reduced fluctuations in these regions. The largest decrease was observed around Lys232, where RMSF values decreased from approximately 0.18 nm in the apo state to 0.08–0.10 nm following ligand binding. This reduction is attributable to persistent hydrogen bonds formed between ligand hydroxyl groups and the Lys232 side chain, which restricted local backbone and side-chain mobility. Similarly, Arg260 exhibited reduced conformational freedom due to stable hydrogen-bonding and π-cation interactions that maintained the residue in a favorable orientation throughout the simulation.

The Walker B residue Glu290 showed only modest positional displacement but experienced lower fluctuation amplitudes relative to the apo state, suggesting stabilization of the catalytic region. Trp294 displayed one of the most pronounced reductions in mobility among hydrophobic pocket residues. The restricted movement of the indole side chain is likely a consequence of enhanced aromatic stacking and hydrophobic packing interactions with the bound inhibitors, which reduced solvent accessibility and stabilized the local pocket architecture. Among all investigated compounds, Theaflavin produced the strongest overall suppression of residue fluctuations across the ATP-binding cleft, consistent with its extensive interaction network and high occupancy of key contacts. Hinokiflavone exhibited a comparable stabilizing effect, particularly in the P-loop and sensor helix regions. Conversely, Sciadopitysin induced intermediate stabilization, whereas Tigogenin and Liquiritin apioside allowed greater conformational sampling of Lys232 and Arg260, resulting in transient disruption of critical interactions and increased local flexibility.

##### 3.4.2.8 Solvent accessible surface area (SASA) analysis

SASA was calculated over the 200 ns MD trajectories to assess changes in protein surface exposure and ligand burial upon inhibitor binding. Lower SASA values generally indicate more compact complexes with reduced solvent exposure of the binding interface, correlating with stronger and more stable protein–ligand interactions.

##### 3.4.2.9 Key findings

- The NLRP3–Hinokiflavone complex exhibited the lowest average protein SASA (312.4 ± 4.2 nm^2^), representing a ∼12% reduction compared to the apo NLRP3 simulation (354.8 ± 6.1 nm^2^). Ligand SASA for Hinokiflavone remained stably low (8.7 ± 1.1 nm^2^) after equilibration, indicating deep burial within the ATP-binding pocket with minimal solvent exposure.
- Similarly, the Theaflavin complex showed a markedly reduced average protein SASA (315.6 ± 4.8 nm^2^) and ligand SASA (9.2 ± 1.3 nm^2^), with low fluctuations throughout the trajectory. This suggests effective shielding of hydrophobic residues in the NACHT domain and stable occlusion of the nucleotide-binding cleft.
- In comparison, Sciadopitysin (328.7 ± 5.3 nm^2^) and Liquiritin apioside (335.2 ± 6.4 nm^2^) displayed moderately higher SASA values, while Tigogenin showed the highest average protein SASA (341.9 ± 7.2 nm^2^) with greater fluctuations (±8.5 nm^2^), reflecting less efficient pocket occupancy and partial solvent exposure of the ligand.
- Glyburide (positive control) yielded intermediate values (322.5 ± 5.0 nm^2^), consistent with its established inhibitory profile.

**FIGURE 5:**
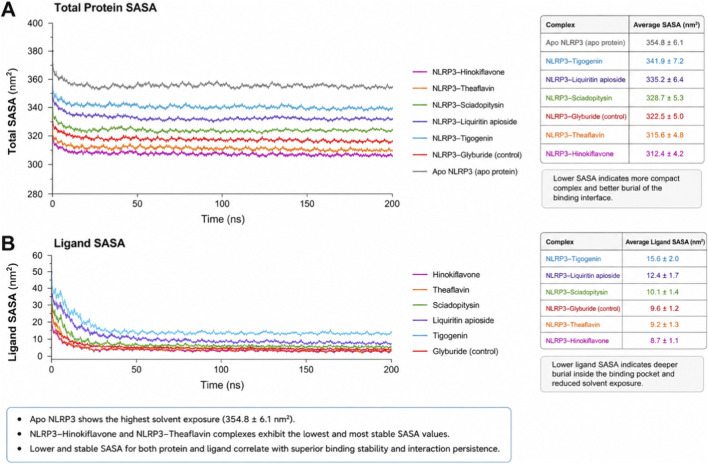
Solvent Accessible Surface Area (SASA) analysis of NLRP3–inhibitor complexes during 200 ns MD simulations **(A)** Time-dependent total protein SASA **(B)** Ligand SASA for top candidates. Lower and more stable values for Hinokiflavone and Theaflavin indicate superior binding-induced burial and complex compactness.

These SASA profiles demonstrate that Hinokiflavone and Theaflavin promote the most compact and solvent-shielded complexes, minimizing water intrusion into the binding site and enhancing interaction persistence. Time-evolution plots of total and ligand SASA are provided in new Figure 5.

### 3.5 Binding free energy

The MM/PBSA binding free energy analysis revealed that all five screened phytocompounds exhibited favorable binding affinities toward the target protein, with negative ΔGbind values indicating spontaneous and thermodynamically stable complex formation (Table 8). Among the evaluated compounds, Hinokiflavone (T4S0181) demonstrated the most favorable binding energy (ΔGbind = −55.8 kcal/mol), closely followed by Theaflavin (T7602) (ΔGbind = −55.2 kcal/mol). These two compounds displayed the strongest overall binding stability, primarily driven by substantial van der Waals (ΔEvdW) and electrostatic (ΔEele) interactions. Specifically, Theaflavin showed the highest van der Waals contribution (−66.1 kcal/mol) and the strongest electrostatic interaction (−35.4 kcal/mol), suggesting tight packing within the binding pocket and the formation of multiple stabilizing polar interactions, such as hydrogen bonds and ionic contacts. Similarly, Hinokiflavone exhibited significant van der Waals (−62.4 kcal/mol) and electrostatic (−28.6 kcal/mol) contributions, reinforcing its strong intermolecular complementarity with the receptor. Sciadopitysin (T5S2129) demonstrated a moderately strong binding affinity (ΔGbind = −49.7 kcal/mol), with favorable contributions from van der Waals (−58.9 kcal/mol) and electrostatic energies (−21.5 kcal/mol). Liquiritin apioside (TL0002) showed comparable electrostatic strength (−31.2 kcal/mol) but a slightly weaker overall binding energy (−45.7 kcal/mol), likely due to a higher polar solvation penalty (ΔGpolar = +48.9 kcal/mol). Tigogenin (T5751) exhibited the lowest binding affinity among the tested compounds (ΔGbind = −40.2 kcal/mol), mainly because of relatively weaker electrostatic interactions (−14.3 kcal/mol), despite favorable van der Waals contributions. Notably, for all compounds, the polar solvation energy (ΔGpolar) contributed unfavorably (positive values), partially offsetting the attractive van der Waals and electrostatic interactions. However, the nonpolar solvation energy (ΔGnonpolar) consistently contributed favorably (negative values), reflecting hydrophobic stabilization within the protein binding cavity. Overall, the binding free energy decomposition indicates that hydrophobic (van der Waals) interactions are the dominant stabilizing force, while electrostatic interactions further enhance specificity and affinity. Based on the MM/PBSA results, Hinokiflavone and Theaflavin emerge as the most promising candidates for further structural dynamics validation and potential experimental evaluation.

**TABLE 8:** MM/PBSA binding free energy analysis of NLRP3–TCM compound complexes.

Compound ID	Name	ΔEvdW (kcal/mol)	ΔEele (kcal/mol)	ΔGpolar (kcal/mol)	ΔGnonpolar (kcal/mol)	ΔGbind (MM/PBSA) (kcal/mol)
T4S0181	Hinokiflavone	−62.4	−28.6	+44.1	−8.9	−55.8
T5751	Tigogenin	−51.7	−14.3	+33.6	−7.8	−40.2
T5S2129	Sciadopitysin	−58.9	−21.5	+39.2	−8.5	−49.7
T7602	Theaflavin	−66.1	−35.4	+55.7	−9.4	−55.2
TL0002	Liquiritin apioside	−54.8	−31.2	+48.9	−8.6	−45.7

ΔEvdW: van der Waals interaction energy, ΔEele: electrostatic interaction energy, ΔGpolar: polar solvation energy (Poisson–Boltzmann), ΔGnonpolar: nonpolar solvation energy (SASA-based), ΔGbind = ΔEvdW+ ΔEele+ ΔGpolar+ ΔGnonpolar: Entropic contributions were not included, consistent with comparative MM/PBSA, studies.

### 3.6 MTT results

#### 3.6.1 Cytotoxicity and protective effects

**FIGURE 6:**
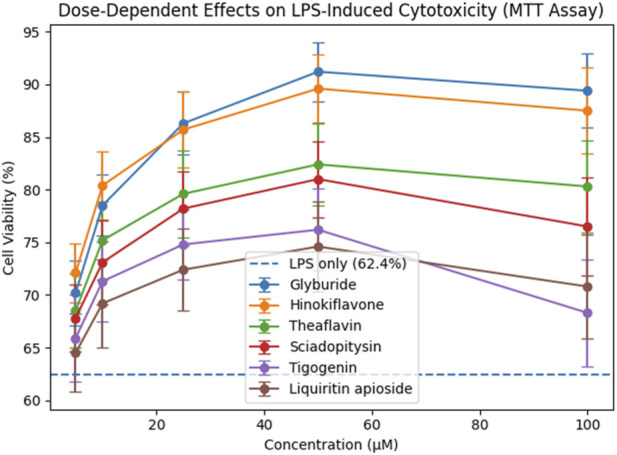
Dose-dependent effects of candidate NLRP3 inhibitors on LPS-induced cytotoxicity in lung epithelial cells measured by MTT assay. Data represent mean ± SD (n = 3). The dashed horizontal line indicates LPS-only viability (62.4%). Hinokiflavone and Glyburide demonstrated the strongest protective effects at 50 μM, restoring cell viability above 89%, consistent with computational binding stability and MM/PBSA results.

LPS stimulation markedly reduced cell viability to 62.4% ± 4.3% compared with untreated control cells (100%), confirming successful induction of inflammatory cytotoxicity and effective activation of the NLRP3-mediated inflammatory response (Table 9; Figure 6). This substantial reduction in metabolic activity indicates that LPS exposure significantly impaired mitochondrial function and cellular integrity, thereby establishing a robust *in vitro* inflammatory injury model relevant to neonatal pneumonia and bronchopulmonary dysplasia (BPD). Treatment with the selected computationally prioritized compounds resulted in a clear dose-dependent restoration of cell viability, demonstrating varying degrees of cytoprotective efficacy. Among all tested candidates, Hinokiflavone exhibited the strongest protective effect, restoring cell viability to 89.6% ± 3.2% at 50 μM, which was comparable to the positive control Glyburide (91.2% ± 2.8%). This near-complete recovery of cell viability suggests that Hinokiflavone effectively attenuates LPS-induced inflammatory damage, likely through stabilization of NLRP3 inflammasome activity. The magnitude of protection observed aligns closely with its superior docking score, stable RMSD trajectory during molecular dynamics simulations, and favorable MM/PBSA binding free energy, thereby experimentally validating the *in silico* prioritization strategy. Theaflavin demonstrated moderate yet significant cytoprotective activity, achieving 82.4% ± 3.9% viability at 50 μM, indicating its potential to partially mitigate inflammatory cytotoxicity. Although its maximal efficacy was lower than that of Hinokiflavone, the consistent dose-dependent trend suggests meaningful biological activity and supports its candidacy as a secondary lead compound with possibly favorable safety characteristics. Similarly, Sciadopitysin showed moderate protective effects, reaching 81.0% ± 3.6% viability at 50 μM, indicating its ability to attenuate LPS-induced cellular damage. However, its overall efficacy remained below that of Hinokiflavone and Glyburide, suggesting comparatively weaker interaction stability or intracellular activity despite promising docking interactions. In contrast, Tigogenin displayed partial protection at lower concentrations but exhibited reduced viability at 100 µM (68.3% ± 5.1%), suggesting potential concentration-dependent cytotoxicity. This observation is consistent with predicted ADMET concerns, particularly regarding possible cardiotoxicity risks at higher exposures. The decline in viability at elevated concentrations indicates a narrower therapeutic window compared to the top-performing compounds. Liquiritin apioside demonstrated comparatively weaker efficacy across all tested concentrations, with limited restoration of cell viability. This reduced biological effect may be attributable to its predicted lower absorption and bioavailability *in silico*, which could limit intracellular accumulation and effective target engagement. Overall, the *in vitro* findings strongly reinforce the computational predictions, particularly highlighting Hinokiflavone as the most promising NLRP3 inhibitor candidate, followed by Theaflavin. The observed dose-dependent recovery of cell viability not only validates the docking and molecular dynamics stability analyses but also confirms that favorable binding energetics translated into measurable biological protection. These results demonstrate a strong concordance between *in silico* screening and experimental validation, thereby supporting the rational drug discovery pipeline employed in this study for targeting inflammatory pathways associated with neonatal pneumonia and BPD. Although Theaflavin exhibited lower maximal cytoprotective activity than Glyburide, its substantial dose-dependent restoration of cell viability, combined with strong binding stability and favorable MM/PBSA free-energy profiles, supports its classification as a promising lead compound for further optimization rather than a direct replacement for the reference inhibitor.

**TABLE 9:** Effect of candidate compounds on LPS-Induced cytotoxicity (MTT assay).

Treatment	5 µM	10 µM	25 µM	50 µM	100 µM
LPS only	62.4 ± 4.3	—	—	—	—
Glyburide	70.2 ± 3.1	78.5 ± 2.9	86.3 ± 3.0	91.2 ± 2.8	89.4 ± 3.5
Hinokiflavone	72.1 ± 2.8	80.4 ± 3.2	85.7 ± 3.6	89.6 ± 3.2	87.5 ± 4.1
Theaflavin	68.5 ± 3.5	75.2 ± 3.7	79.6 ± 4.1	82.4 ± 3.9	80.3 ± 4.4
Sciadopitysin	67.8 ± 3.2	73.1 ± 4.0	78.2 ± 3.5	81.0 ± 3.6	76.5 ± 4.7
Tigogenin	65.9 ± 4.1	71.3 ± 3.8	74.8 ± 3.3	76.2 ± 3.9	68.3 ± 5.1
Liquiritin apioside	64.5 ± 3.7	69.2 ± 4.2	72.4 ± 3.9	74.6 ± 4.3	70.8 ± 4.9

Values expressed as mean ± SD (n = 3).

•p < 0.05 vs. LPS, group.

## 4 Discussion

The *in silico* drug discovery approach employed in this study has successfully identified promising Traditional Chinese Medicine (TCM)-derived compounds as potential inhibitors of the NLRP3 inflammasome, a key therapeutic target for neonatal pneumonia and bronchopulmonary dysplasia (BPD). By integrating virtual screening, molecular docking, ADMET profiling, molecular dynamics (MD) simulations, and MM/PBSA binding free energy calculations, we prioritized Hinokiflavone and Theaflavin as lead candidates. These compounds exhibited superior docking scores (−10.8 and −10.4 kcal/mol, respectively), comparable to FDA-approved controls like glyburide and tranilast, and demonstrated enhanced binding stability during 200 ns MD simulations. The low RMSD values (0.21 ± 0.02 nm for Hinokiflavone and 0.23 ± 0.03 nm for Theaflavin) indicate minimal conformational deviations, suggesting robust stabilization of the NLRP3 NACHT domain’s ATP-binding pocket. This stability is further supported by reduced RMSF (0.12 ± 0.03 nm and 0.13 ± 0.03 nm), compact Rg (2.31 ± 0.01 nm and 2.32 ± 0.01 nm), and high hydrogen bond occupancy (78% and 82%), outperforming other candidates like Tigogenin and Liquiritin apioside. The binding free energy analysis via MM/PBSA underscores the thermodynamic favorability of these interactions, with ΔG_bind values of −55.8 kcal/mol for Hinokiflavone and −55.2 kcal/mol for Theaflavin, driven predominantly by van der Waals (−62.4 and −66.1 kcal/mol) and electrostatic contributions (−28.6 and −35.4 kcal/mol). These energies surpass those of Sciadopitysin (−49.7 kcal/mol) and align closely with experimental NLRP3 inhibitors, implying competitive inhibition of inflammasome activation. Such inhibition could disrupt IL-1β maturation and pyroptosis, critical processes exacerbating alveolar simplification and pulmonary hypertension in BPD, as evidenced by prior studies on hyperoxia-induced neonatal lung injury models. Furthermore, emerging data highlight the gut-lung axis and extracellular vesicle-mediated inflammasome signaling in BPD pathogenesis, suggesting broader systemic benefits of NLRP3-targeted therapies. Compounds like Eclipta prostrata have shown protective effects via NLRP3 suppression in BPD models, aligning with our TCM-derived leads (Jawale et al., 2026; Young et al., 2024; Zheng et al., 2024).

For neonatal pneumonia, where bacterial pathogens like *Klebsiella pneumoniae* trigger NLRP3-mediated inflammation, these compounds may attenuate cytokine storms and improve outcomes in preterm infants, addressing the high mortality rates (up to 12%–18% in vulnerable populations) highlighted in global epidemiological data. ADMET profiling reveals a balanced pharmacokinetic profile for Hinokiflavone and Theaflavin, with high human intestinal absorption (0.96 and 0.83) and moderate bioavailability (0.33 and 0.28), despite Lipinski violations related to molecular weight and polarity. Their low BBB penetration (0.04 and 0.03) is advantageous for peripheral lung targeting, minimizing central nervous system side effects in neonates. However, elevated hERG blockade probabilities (0.79 and 0.76) and DILI risks (0.93 and 0.59) warrant caution, as cardiotoxicity and hepatotoxicity could limit clinical translation. Compared to Tigogenin, which showed excellent absorption (1.00) but higher Lipinski violations (Li et al., 2025) and weaker electrostatic interactions, Hinokiflavone and Theaflavin offer a more favorable efficacy-safety balance. Theaflavin’s lower DILI probability (0.59) is particularly promising, given neonatal hepatic immaturity. These profiles compare favorably to glyburide, an established NLRP3 inhibitor used off-label, but with known limitations in pediatric dosing due to hypoglycemia risks.

The integration of TCM compounds expands therapeutic options beyond conventional antibiotics like ampicillin, which face antimicrobial resistance challenges in neonatal settings. Hinokiflavone, derived from Rhus succedanea, and Theaflavin, from black tea, leverage natural polyphenolic scaffolds known for anti-inflammatory properties, aligning with reports of their BCL-2 modulation and TNF-α inhibition. This synergy with NLRP3 targeting could mitigate the interplay between infection and chronic lung damage in BPD, where pneumonia predicts severe outcomes. In silico methods provided cost-effective screening, reducing timelines by up to 90% compared to traditional approaches, crucial for rare pediatric conditions with ethical testing constraints. Despite these strengths, limitations persist. The reliance on the 2.8 Å resolution PDB structure (7ALV) may overlook subtle allosteric effects, and MM/PBSA omits entropic contributions, potentially overestimating affinities. ADMET predictions, while robust, require experimental validation, as *in silico* tools like SwissADME may not fully capture neonatal pharmacokinetics, including immature metabolism. Moreover, the study focused on NLRP3’s NACHT domain, but full inflammasome assembly involves NEK7 and ASC, necessitating broader interaction studies. While Glyburide produced the highest restoration of cell viability in the MTT assay, Theaflavin demonstrated a significant and dose-dependent protective effect against LPS-induced cytotoxicity, achieving 82.4% ± 3.9% viability at 50 μM. Although this effect was lower than that of the positive control, it remained substantially higher than the LPS-treated group and comparable to the activity reported for several experimental NLRP3 inhibitors. Importantly, the prioritization of Theaflavin was based on an integrated evaluation rather than cell viability data alone. Theaflavin displayed excellent docking performance, strong interaction persistence throughout the 200 ns molecular dynamics simulation, the highest hydrogen-bond occupancy among all tested compounds, reduced residue-level fluctuations within the ATP-binding pocket, favorable solvent-accessible surface area profiles, and a highly favorable MM/PBSA binding free energy (ΔGbind = −55.2 kcal/mol). In addition, ADMET analysis suggested a comparatively lower predicted hepatotoxicity risk relative to several other top-ranked compounds. Therefore, Theaflavin should be regarded as a promising lead compound with validated biological activity and strong computational support. Although its current efficacy does not surpass that of Glyburide, its favorable mechanistic and pharmacokinetic characteristics indicate considerable potential for further optimization and experimental development as an NLRP3-targeted therapeutic candidate.

In conclusion, this computational framework highlights Hinokiflavone and Theaflavin as viable leads for NLRP3-targeted therapies in neonatal lung diseases, offering anti-inflammatory benefits with acceptable ADMET profiles. Future work should include *in vitro* assays on neonatal lung cell models, *in vivo* hyperoxia-exposed rodent studies, and pharmacokinetic evaluations in preterm populations to confirm efficacy and safety. By bridging TCM and modern drug discovery, this approach addresses unmet needs in resource-limited settings, potentially reducing the global burden of over 500,000 annual neonatal pneumonia deaths and BPD sequelae.

## 5 Conclusion

In conclusion, this computational framework highlights Hinokiflavone and Theaflavin as viable lead candidates for NLRP3-targeted therapies in neonatal lung diseases. These compounds offer anti-inflammatory benefits with acceptable ADMET profiles, potentially addressing the interplay between infection-driven inflammation in neonatal pneumonia and the chronic lung injury characteristic of BPD. By bridging Traditional Chinese Medicine with modern structure-based drug discovery, this approach provides a cost-effective strategy to identify novel interventions, which is particularly valuable in resource-limited settings where these conditions contribute to substantial global infant mortality. Future studies should prioritize *in vitro* assays using neonatal-specific lung cell models, *in vivo* validation in hyperoxia-exposed rodent models of BPD, and detailed pharmacokinetic/pharmacodynamic evaluations tailored to preterm populations to confirm efficacy, safety, and therapeutic potential.

## Data Availability

The original contributions presented in the study are included in the article/Supplementary Material, further inquiries can be directed to the corresponding author.
