# Supplementary material for: In silico drug discovery and molecular dynamics simulation for targeting neonatal pneumonia and bronchopulmonary dysplasia
Source: Front Chem. 2026 Jun 30;14:1859262. doi: 10.3389/fchem.2026.1859262 (PMC13366417; doi:10.3389/fchem.2026.1859262)
Supplement: Supplementary file 1 [file Supplementaryfile1.docx]

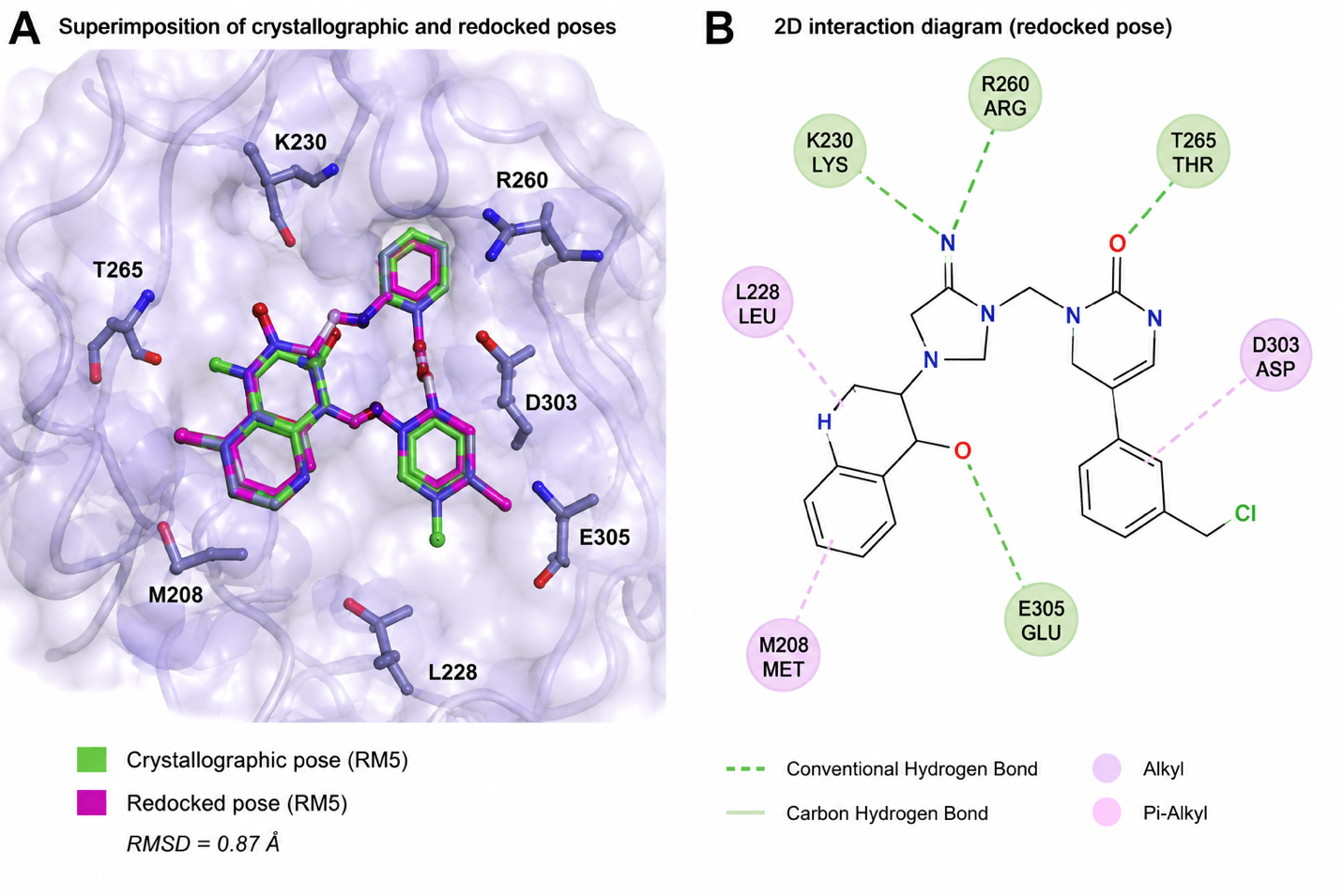


**Figure S1.** Validation of the docking protocol by redocking the co-crystallized inhibitor RM5 into the NLRP3 NACHT domain (PDB 7ALV). (A) Superimposition of the crystallographic pose (green) and the best redocked pose (magenta), RMSD = 0.87 Å. (B) 2D interaction diagram showing conserved hydrogen bonds and hydrophobic interactions reproduced in the redocked pose.
