# Supplementary material for: In silico drug discovery and molecular dynamics simulation for targeting neonatal pneumonia and bronchopulmonary dysplasia
Source: Front Chem. 2026 Jun 30;14:1859262. doi: 10.3389/fchem.2026.1859262 (PMC13366417; doi:10.3389/fchem.2026.1859262)
Supplement: Supplementary file 2 [file Table1.docx]

**Table S1. Docking Score and Physicochemical Properties of Screened DrugBank (FDA-approved) Compounds.**

| **ID** | **Name** | **Formula** | **CAS** | **Score (kcal/mol)** | **MW (Da)** | **HBD** | **HBA** | **RB** | **NOA** | **Rings** | **LogP** |
| --- | --- | --- | --- | --- | --- | --- | --- | --- | --- | --- | --- |
| DB01126 | Dutasteride | C27H30F6N2O2 | 164656-23-9 | -10.8 | 528.53 | 2 | 2 | 3 | 4 | 5 | 6.5 |
| DB01419 | Antrafenine | C30H26F6N4O2 | 55300-29-3 | -10.7 | 588.54 | 1 | 2 | 8 | 6 | 5 | 7.5 |
| DB11799 | Bictegravir | C21H18F3N3O5 | 1611493-60-7 | -10.3 | 449.39 | 2 | 4 | 5 | 8 | 5 | 3.5 |
| DB00549 | Zafirlukast | C31H33N3O6S | 107753-78-6 | -10.2 | 575.68 | 2 | 4 | 11 | 9 | 5 | 5.5 |
| DB09280 | Lumacaftor | C24H18F2N2O5 | 936727-05-8 | -10.2 | 452.41 | 2 | 4 | 7 | 7 | 5 | 4.4 |
| DB12457 | Rimegepant | C28H28F2N6O3 | 1289023-67-1 | -10.1 | 534.57 | 1 | 4 | 5 | 9 | 6 | 3.5 |
| DB00210 | Adapalene | C28H28O3 | 106685-40-9 | -10.1 | 412.52 | 1 | 2 | 5 | 3 | 6 | 7.7 |
| DB08815 | Lurasidone | C28H36N4O2S | 367514-87-2 | -10.0 | 492.68 | 0 | 3 | 5 | 6 | 7 | 5.3 |
| DB08912 | Dabrafenib | C23H20F3N5O2S2 | 1195765-45-7 | -9.9 | 519.56 | 2 | 5 | 6 | 7 | 4 | 4.7 |
| DB09291 | Rolapitant | C25H26F6N2O2 | 552292-08-7 | -9.9 | 500.49 | 2 | 1 | 5 | 4 | 4 | 4.4 |
| DB04835 | Maraviroc | C29H41F2N5O | 376348-65-1 | -9.9 | 513.67 | 1 | 3 | 9 | 6 | 5 | 5.1 |
| DB06210 | Eltrombopag | C25H22N4O4 | 496775-61-2 | -9.9 | 442.47 | 3 | 4 | 7 | 8 | 4 | 4.7 |
| DB11262 | Bisoctrizole | C41H50N6O2 | 103597-45-1 | -9.8 | 658.89 | 2 | 6 | 12 | 8 | 6 | 12.7 |
| DB12020 | Tecovirimat | C19H15F3N2O3 | 869572-92-9 | -9.8 | 376.34 | 1 | 3 | 3 | 5 | 5 | 2.6 |
| DB01100 | Pimozide | C28H29F2N3O | 2062-78-4 | -9.8 | 461.55 | 0 | 1 | 7 | 4 | 5 | 6.9 |
| DB09074 | Olaparib | C24H23FN4O3 | 763113-22-0 | -9.8 | 434.46 | 0 | 4 | 6 | 7 | 5 | 3.5 |
| DB13953 | Estradiol benzoate | C25H28O3 | 50-50-0 | -9.8 | 376.49 | 1 | 2 | 4 | 3 | 5 | 5.6 |
| DB09335 | Alatrofloxacin | C26H25F3N6O5 | 146961-76-4 | -9.8 | 558.52 | 4 | 6 | 10 | 11 | 5 | 0.9 |
| DB15035 | Zanubrutinib | C27H29N5O3 | 1691249-45-2 | -9.8 | 471.56 | 2 | 3 | 7 | 8 | 5 | 3.5 |
| DB13520 | Metergoline | C25H29N3O2 | 17692-51-2 | -9.8 | 403.53 | 1 | 1 | 6 | 5 | 5 | 3.7 |
| DB11652 | Tucatinib | C26H24N8O2 | 937263-43-9 | -9.8 | 480.53 | 2 | 4 | 6 | 10 | 6 | 3.9 |
| DB12887 | Tazemetostat | C34H44N4O4 | 1403254-99-8 | -7.7 | 572.75 | 1 | 2 | 10 | 8 | 5 | 6.0 |

**Table S2. Docking Scores and Physicochemical Properties of Screened TCM Compounds**

| **ID** | **Name** | **Formula** | **CAS** | **Docking Score (kcal/mol)** | **MW (Da)** | **HBD** | **HBA** | **RB** | **N/O Atoms** | **Rings** | **logP** | **Herbal Sources** |
| --- | --- | --- | --- | --- | --- | --- | --- | --- | --- | --- | --- | --- |
| T4S0181 | Hinokiflavone | C30H18O10 | 19202-36-9 | -10.8 | 538.5 | 5 | 7 | 9 | 10 | 6 | 3.7 | 侧柏叶 |
| T5751 | Tigogenin | C27H44O3 | 77-60-1 | -10.4 | 416.64 | 1 | 1 | 1 | 3 | 6 | 6.4 | 丹参; 知母; 蒺藜; 酸浆 |
| T5S2129 | Sciadopitysin | C33H24O10 | 521-34-6 | -10.4 | 580.54 | 3 | 5 | 9 | 10 | 6 | 4.4 | 白果 |
| T7602 | Theaflavin | C29H24O12 | 46705-7 | -10.4 | 564.49 | 9 | 10 | 11 | 12 | 6 | -0.6 | 山茶 |
| TL0002 | Liquiritin apioside | C26H30O13 | 74639-14-8 | -10.0 | 550.51 | 7 | 8 | 14 | 13 | 5 | -0.8 | 甘草; 绵萆薢 |
| T4036 | Solasodine | C27H43NO2 | 126-17-0 | -10.0 | 413.65 | 2 | 1 | 1 | 3 | 6 | 5.3 | 白毛藤; 辣椒 |
| TN1877 | Lonicerin | C27H30O15 | 25694-72-8 | -9.8 | 594.50 | 9 | 10 | 15 | 15 | 5 | -1.3 | 化橘红; 金银花 |
| TN6712 | Yibeissine | C27H41NO4 | 143502-51-6 | -9.7 | 443.60 | 3 | 3 | 2 | 5 | 6 | 1.6 | 伊贝母 |
| T5S0106 | Peimisine | C27H41NO3 | 19773-24-1 | -9.7 | 427.62 | 2 | 2 | 1 | 4 | 6 | 2.7 | 川贝母; 浙贝母 |
| T2912 | Ergosterol | C28H44O | 57-87-4 | -9.7 | 396.65 | 1 | 1 | 5 | 1 | 4 | 7.4 | 云芝; 冬虫夏草 |
| TN1166 | 1,3,6-Tri-O-galloyl-β-D-glucose | C27H24O18 | 18483-17-5 | -9.7 | 636.50 | 11 | 14 | 21 | 18 | 4 | 0.3 | 牡丹皮 |
| TN1860 | Licoflavone B | C25H26O4 | 91433-17-9 | -9.7 | 390.47 | 2 | 3 | 7 | 4 | 3 | 5.5 | 杜仲; 甘草 |
| T1633 | β-Carotene | C40H56 | 7235-40-7 | -9.7 | 536.87 | 0 | 0 | 10 | 0 | 2 | 13.6 | 枸杞子; 辣椒 |
| T8184 | Fucosterol | C29H48O | 17605-67-3 | -9.6 | 412.70 | 1 | 1 | 6 | 1 | 4 | 8.8 | 山药; 海藻 |
| T6S0141 | Corylin | C20H16O4 | 53947-92-5 | -9.6 | 320.30 | 1 | 2 | 2 | 4 | 4 | 2.9 | 葛根; 补骨脂 |
| T5S1103 | Isoliensinine | C37H42N2O6 | 6817-41-0 | -9.6 | 610.75 | 2 | 2 | 11 | 8 | 6 | 6.3 | 莲子 |
